# Mitochondrial-targeting Mn_3_O_4_/UIO-TPP nanozyme scavenge ROS to restore mitochondrial function for osteoarthritis therapy

**DOI:** 10.1093/rb/rbad078

**Published:** 2023-09-01

**Authors:** Shengqing Zhang, Jinhong Cai, Yi Yao, Lanli Huang, Li Zheng, Jinmin Zhao

**Affiliations:** Collaborative Innovation Centre of Regenerative Medicine and Medical Bioresource Development and Application Co-constructed by the Province and Ministry, The First Affiliated Hospital of Guangxi Medical University, Nanning 530021, China; Guangxi Engineering Center in Biomedical Materials for Tissue and Organ Regeneration, The First Affiliated Hospital of Guangxi Medical University, Nanning 530021, China; Collaborative Innovation Centre of Regenerative Medicine and Medical Bioresource Development and Application Co-constructed by the Province and Ministry, The First Affiliated Hospital of Guangxi Medical University, Nanning 530021, China; Guangxi Engineering Center in Biomedical Materials for Tissue and Organ Regeneration, The First Affiliated Hospital of Guangxi Medical University, Nanning 530021, China; Guangxi Engineering Center in Biomedical Materials for Tissue and Organ Regeneration, The First Affiliated Hospital of Guangxi Medical University, Nanning 530021, China; Life Sciences Institute, Guangxi Medical University, Nanning 530021, China; Guangxi Engineering Center in Biomedical Materials for Tissue and Organ Regeneration, The First Affiliated Hospital of Guangxi Medical University, Nanning 530021, China; Collaborative Innovation Centre of Regenerative Medicine and Medical Bioresource Development and Application Co-constructed by the Province and Ministry, The First Affiliated Hospital of Guangxi Medical University, Nanning 530021, China; Guangxi Engineering Center in Biomedical Materials for Tissue and Organ Regeneration, The First Affiliated Hospital of Guangxi Medical University, Nanning 530021, China; Collaborative Innovation Centre of Regenerative Medicine and Medical Bioresource Development and Application Co-constructed by the Province and Ministry, The First Affiliated Hospital of Guangxi Medical University, Nanning 530021, China; Guangxi Engineering Center in Biomedical Materials for Tissue and Organ Regeneration, The First Affiliated Hospital of Guangxi Medical University, Nanning 530021, China; Department of Orthopedics, The First Affiliated Hospital of Guangxi Medical University, Nanning 530021, China

**Keywords:** Mn_3_O_4_, nanozyme, ROS scavenging, mitochondrial-targeting, osteoarthritis

## Abstract

Excessive reactive oxygen species (ROS)-induced mitochondrial damage has impact on osteoarthritis (OA). Nanozyme mimics as natural enzyme alternatives to scavenge excessive ROS has offered a promising strategy for OA therapy. Herein, we reported a novel mitochondrial-targeting Mn_3_O_4_/UIO-TPP nanozyme using metal-organic frameworks with loaded Mn_3_O_4_ as the enzyme-like active core combining mitochondria-targeting triphenylphosphine (TPP) groups to serve as ROS scavengers for therapy of OA. With sequential catalysis of superoxide dismutase-like, catalase (CAT)-like, and hydroxyl radical (·OH) scavenging potentials, the nanozyme can target mitochondria by crossing subcellular barriers to effectively eliminate ROS to restore mitochondrial function and inhibit inflammation and chondrocyte apoptosis. It also has favorable biocompatibility and biosafety. Based on anterior cruciate ligament transection-induced OA joint models, this mitochondrial-targeting nanozyme effectively mitigated the inflammatory response with the Pelletier score reduction of 49.9% after 8-week therapy. This study offers a prospective approach to the design of nanomedicines for ROS-related diseases.

## Graphical Abstract

**Figure:**
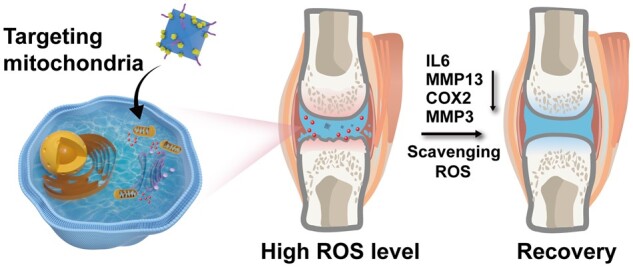

## Introduction

Osteoarthritis (OA), characterized by pain, joint inflammation, articular cartilage defects and limited joint movement, was one of the prevailing joint disorders across the world, threatening public health [1–3]. Increasing evidence showed that mitochondrial dysfunction caused by reactive oxygen species (ROS)-induced mitochondrial DNA (mtDNA) damage contributes to the progression of OA, involved in PI3K-Akt, Caspase and matrix metalloproteinases (MMPs) signaling pathways in chondrocytes, as well as chondrocyte apoptosis [4–9]. Antioxidants for scavenging ROS such as superoxide dismutase (SOD), Vitamins, Coenzyme Q10, Polyphenol and Polysaccharides, have been reported to reduce oxidative stress and improve the histological score of the cartilages, were regarded as a prospective approach for scavenging ROS and further therapy of OA. However, most ROS scavengers lack specific targeting and have weak ROS scavenging efficiency with short retention time *in vivo* [10, 11], leading to unfavorable therapeutic effects.

Recently, nanozymes like noble metals, metal oxides, carbides etc. with the abilities to scavenge ROS have been extensively used in inflammatory diseases [12–15], with superior stability and ease of availability in comparison to natural enzymes. Comparatively, nanozymes have better enzyme degradation resistance *in vivo* [16], which could stay longer in tissues and improve the therapeutic efficacy, reducing repeated administration and the risk of infection during treatment [17]. Previous researches have confirmed that nanoscale Mn_3_O_4_ could present multi-enzyme-like behavior, including SOD-like, catalase (CAT)-like and ·OH scavenging activities in the medication of ROS-related diseases, including inflammatory bowel diseases, acute kidney injury, cardiovascular and cerebrovascular diseases, and other tissue inflammation [18–21]. Due to its mixed oxidation states and high stability in redox reaction, Mn_3_O_4_ nanoparticles (NPs) could achieve high stability and show superiority in eliminating ROS and showed better therapeutic efficacy than clinical medicals as Mugesh et al. [22] and Wei et al. [19] reported. However, Mn_3_O_4_ NPs were easy to agglomerate and sediment, leading to loss of catalytic properties and increase of cytotoxicity [23, 24], discounting their therapeutic effect on ROS-related diseases.

By using carriers such as metal-organic frameworks (MOFs), transition metal carbide (MXene), and carbon dots, were proven effective to improve catalytic activity of nanozymes due to the electronic interaction between nanozymes and carriers [25]. In favor of anchoring functional molecules, MOFs were a kind of newly porous crystalline material with facile synthesis, intrinsic biodegradability, tunable pore cavity and large surface area [26]. These characteristics made MOFs potentially suitable for limiting the movement and promoting the interaction of NPs, ensuring the smooth diffusion of reactants by uniform dispersion [27, 28]. As a uniform-shaped MOFs with the highest coordination number of organic ligands and metal clusters, dense unit arrangement and strong Zr–O bond, the UIO66 series have superior hydrothermal, mechanical and chemical stability [29–31]. This excellent stability benefits the drug bioavailability *in vivo*. It has been reported that UIO66 carriers for Pt, Au and Ag NPs have greatly improved the bioactivity for tumor treatment and wound healing [24, 32, 33]. Thus, the introduction of UIO66 series MOFs may increase the catalysis and decrease the cytotoxicity of Mn_3_O_4_ NPs by preventing the rapid release of Mn ions [19, 34].

**Figure 1.**
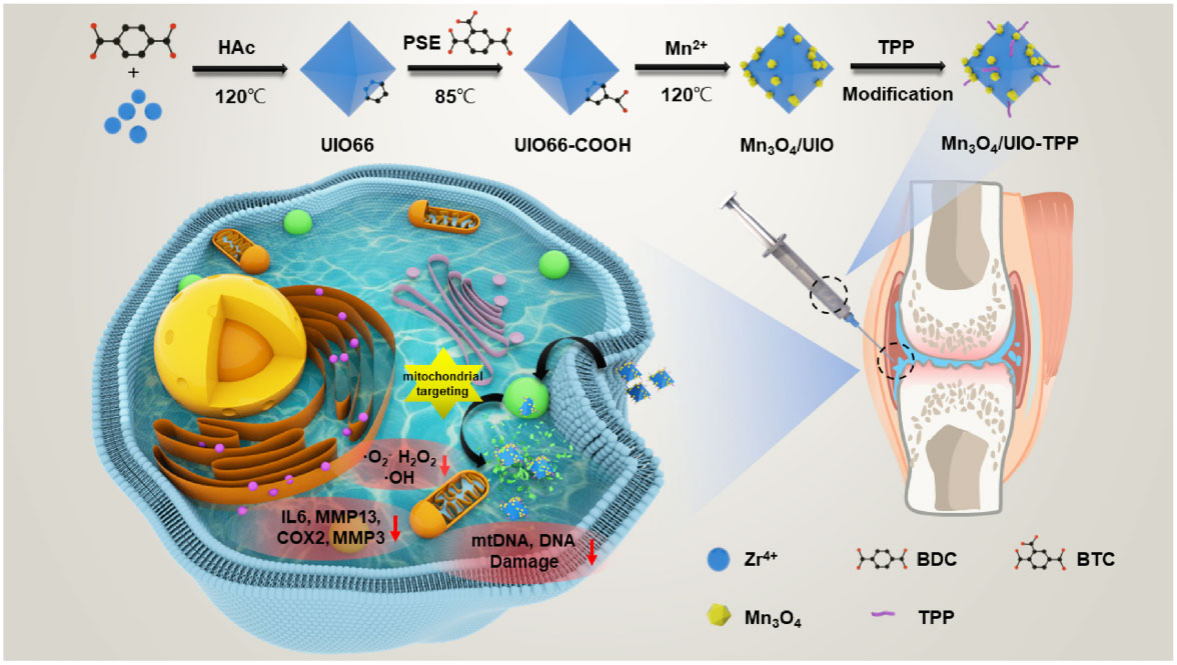
Fabrication route of Mn_3_O_4_/UIO-TPP and lowering ROS levels, reducing mtDNA/DNA damage, and anti-inflammatory for therapy of OA by mitochondrial-targeting.

In our study, a multifunctional Mn_3_O_4_/UIO-TPP nanozyme (Figure 1) was developed by grafting Mn_3_O_4_ NPs with UIO66 series MOFs using a hydrothermal method followed by facile conjugation with mitochondria-targeting triphenylphosphine (TPP) groups, in an attempt to scavenge mitochondrion-derived ROS to restore mitochondrial damage for OA therapy. The modified MOFs facilitate subcellular targeting at the lesion site through rapid localization of diseased cells and precisely modulation of organelle function [35], with an efficient mitochondria ROS scavenging efficiency to enhance therapeutic effect. This study may offer a promising strategy for effective mitochondrial-targeting therapy of ROS-related diseases.

## Materials and methods

### Chemicals

Methanol (AR) and Ethanol (AR) were bought from Sinopharm Chemical Reagent Co., Ltd (Shanghai, China). Acetic acid (HAc, 99.5%), N,N-dimethylformamide (DMF, 99.5%), Zirconium(IV) chloride (ZrCl_4_, 99.9%), 1,4-benzenetricarboxylic acid (BDC, 99%), 1,2,4-benzenetricarboxylic acid (BTC, 98%) and manganese acetate tetrahydrate (Mn(CH_3_COO)_2_·4H_2_O, 99.9%) were bought from Aladdin (Shanghai, China).

(3-Aminopropyl) (triphenyl) phosphonium bromide (NH_2_-TPP, 95%), N-Hydroxysuccinimide (NHS, 98%) and 1-(3-Dimethylaminopropyl)-3-ethylcarbodiimide hydrochloride (EDC, 95%) were bought from Macklin (Shanghai, China).

### Synthesis of Mn_3_O_4_ NPs

Mn_3_O_4_ NPs synthesizing was referred from the method in previous work [36] with slightly modified. According to a routine procedure, 613 mg Mn(CH_3_COO)_2_·4H_2_O was added into 30 ml ethanol and heated up to 120°C (5°C/min) for 24 h in an autoclave after fully dissolved under ultrasound. Once naturally cooled to ambient temperature, the Mn_3_O_4_ NPs were subsequently obtained by centrifugation and dried after 12 h in a 60°C vacuum chamber.

### Synthesis of UIO66-COOH

The method for synthesizing precursor UIO66 was referred from previous work [37] with slightly modified. According to a routine procedure, 160 mg ZrCl_4_ and 114 mg BDC were added into a degassed solution (40 ml DMF and 1.2 ml HAc) and heated up to 120°C (5°C/min) for 24 h in an autoclave after fully dissolved under ultrasound. Once naturally cooled to ambient temperature, centrifugated to collect and soaked the precipitates into methanol for three days, then dried after 12 h in a 60°C vacuum chamber.

The obtained UIO66 was carboxylated by a post-synthesis exchange method referred from previous work [38, 39] with slightly modified. Typically, 105 mg of 1,2,4-benzene tricarboxylic acid was dissolved it in 2 ml of 4% KOH solution, then the solution pH was adjusted to 7 ± 0.2 with 1 M HCl with an additional 28 mg UIO66 and 0.5 ml DMF. The obtained solution was next heated up to 85°C (5°C/min) for 24 h in an autoclave after ultrasonic dispersion for 30 min. Once naturally cooling to ambient temperature, centrifugated to collect and soaked the precipitates into methanol for three days, then dried after 12 h in a 60°C vacuum chamber.

### Synthesis of Mn_3_O_4_/UIO nanozymes

Typically, 32 mg Mn(CH_3_COO)_2_·4H_2_O and 100 mg UIO66-COOH were added into 20 ml ethanol. The obtained solution was next ultrasonic dispersion for 15 min, and then heated up to 120°C (5°C/min) for 24 h in an autoclave. Once naturally cooled to ambient temperature, centrifugated to collect and washed the precipitates with methanol three times, then dried after 12 h in a 60°C vacuum chamber.

To compare the effect of Mn_3_O_4_ content on nanozyme, Mn_3_O_4_/UIO nanozymes with lower Mn_3_O_4_ content were also synthesized by adjusting the dosage of Mn(CH_3_COO)_2_·4H_2_O to 4 mg or 16 mg while other procedures remain the same.

These three samples were named Mn_3_O_4_/UIO1, Mn_3_O_4_/UIO2 and Mn_3_O_4_/UIO3, according to the feed ratio of Mn(CH_3_COO)_2_·4H_2_O from low to high. The brief reaction conditions were shown in Supplementary Table S1.

### TPP/FITC/Cy5.5 conjugation

Mn_3_O_4_/UIO3 nanozyme was conjugated with TPP via the reaction between –NH_2_ and –NHS ester [40].

For TPP conjugation, 20 mg Mn_3_O_4_/UIO3 nanozyme was ultrasonically dispersed in 20 ml ethanol in a flask. The obtained solution was next stirred at ambient temperature for 20 min with an additional 9.59 mg EDC and 4.95 mg NHS. Then added 20 mg TPP-NH_2_ into the solution and stirred for another 24 h at ambient temperature. Once centrifugated to collect and washed with methanol three times, the product was dried after 12 h in a 60°C vacuum chamber and named Mn_3_O_4_/UIO-TPP.

Moreover, the FITC-labeled Mn_3_O_4_/UIO-TPP or Mn_3_O_4_/UIO and Cy5.5-labeled Mn_3_O_4_/UIO-TPP were obtained by reacting with 5-FITC cadaverine (AAT Bioquest, USA) and Cy5.5-amine (Duofluor, China), respectively.

### Characterization

These NPs were respectively characterized via accelerated surface area and porosimetry system (Micromeritics ASAP-2020, USA), Fourier transform infrared spectrometer (FTIR, IRAffinity-1S, Japan), inductively coupled plasma optical emission spectroscopy (ICP-OES, Optima 8000, USA), Raman spectroscopy (JASCO NRS-4500, Japan), thermogravimetric analysis (TGA, Star449, Germany), transmission electron microscopy (TEM, Talos F200X, USA) with energy dispersive X-ray spectroscopy (EDX), X-ray photoelectron spectroscopy (XPS, ESCALAB 250Xi, USA) and X-ray diffraction (XRD, MiniFlex 600, Japan). The dynamic light scattering (DLS) and Zeta potential of these NPs in PBS buffer were measured via Zetasizer Nano ZS (Malvern Panalytical, UK) as well.

### *In vitro* enzyme-like activity

The SOD-like activity was measured, which was the superoxide (·$O_{2}^{-}$) scavenging ability of Mn_3_O_4_/UIO nanozymes, by using a SOD activity assay kit (Sigma-Aldrich, USA). Typically, 20 μl sample solution (0, 5, 10, 20, 50, 100 μg/ml) was incubated for 20 min at 37°C after being mixed with 200 μl WST-8/enzyme working solution mix, and was recorded via a microplate reader (Thermo Fisher, USA) at the absorbance of 450 nm based on the manufacturer’s guidelines.

The CAT-like activity was measured, which was the H_2_O_2_ scavenging ability of Mn_3_O_4_/UIO nanozymes, by using a CAT activity assay kit (Beyotime, China). Typically, 10 μl 250 mM H_2_O_2_ and 40 μl sample solution were incubated for 5 min at 25°C after being mixed with a working solution mix, then took 10 μl of this reacted solution and added into a chromogenic working solution, and was recorded after incubated for 15 min at 25°C via a microplate reader at the absorbance of 520 nm based on the manufacturer’s guidelines.

The ·OH scavenging ability of Mn_3_O_4_/UIO nanozymes was measured by using a ·OH detection kit (Solarbio, China). Typically, 15 μl sample solution was added into 140 μl working solution mix and incubated for 20 min at 37°C, then took 100 μl supernatant and recorded via a microplate reader at the absorbance of 536 nm based on the manufacturer’s guidelines.

Additionally, electron spin resonance (ESR, Bruker A300, Germany) signals were also measured to evaluate the ·$O_{2}^{-}$, H_2_O_2_ and ·OH scavenging ability of Mn_3_O_4_/UIO nanozymes.

### Extraction and culture of chondrocytes

The joint tissues of male Sprague-Dawley (SD) rats within three days of birth were used for harvesting chondrocytes. The harvested chondrocytes were cultured in fresh Dulbecco modified eagle medium (DMEM, Gibco, USA) with an additional 10% (V/V) fetal bovine serum (Sijiqing, China) and 1% penicillin/streptomycin (Solarbio). Refreshed the cultured medium every 72 h, and the chondrocytes were sub-cultured or collected when reaching 75–85% density for further the further use of experiments.

### Cell cytotoxicity assay

The cytotoxicity of NPs (UIO66-COOH, Mn_3_O_4_/UIO1, Mn_3_O_4_/UIO2 and Mn_3_O_4_/UIO3) was measured by using a Cell Counting Kit-8 (CCK-8, Dojindo, Japan) assay. Briefly, chondrocytes in 96-well plates with a density of 5000 cells/well were incubated with fresh medium mixed with NPs with multiple concentrations (0, 5, 10, 20, 50, 100, 200 μg/ml). The medium was subsequently discarded after 24 h, and the cells were incubated in 100 μl medium (10% CCK-8) for another 1 h after washing three times with PBS buffer. The absorbance was recorded via a microplate reader at 450 nm.

### Live and dead staining assay

Chondrocytes in six-well plates with a density of 2 × 10^5^ cells/well were cultured overnight. Then, 20 μg/ml of UIO66-COOH and Mn_3_O_4_/UIOs were, respectively, added into the medium. The cells were stained with Calcein-AM/PI (Sigma-Aldrich, USA) based on the manufacturer’s guidelines when the 24 h incubation finished, and were washed with PBS buffer three times before being captured via a fluorescence microscope (Olympus BX53, Japan). Besides, the survival of chondrocytes under oxidative stress was also evaluated. Six-well plates with a density of 2 × 10^5^ cells/well of chondrocytes were incubated with 200 μM H_2_O_2_ for 24 h to establish the cell model with oxidative stress damage. Then, 20 μg/ml of UIO66-COOH, Mn_3_O_4_/UIO3 and Mn_3_O_4_/UIO-TPP were, respectively, added into the medium. The cells were stained with Calcein-AM/PI when the 24 h incubation finished and were washed with PBS buffer three times before being captured.

### Lysosomal escape and mitochondrial-targeting

Chondrocytes in confocal dishes with a density of 1 × 10^5^ cells/dish were cultured overnight. Medium with additional FITC-labeled Mn_3_O_4_/UIO-TPP and FITC-labeled Mn_3_O_4_/UIO3 nanozymes (20 μg/ml) were, respectively, replaced. After 3, 6 and 12 h incubation, the medium was refreshed and the chondrocytes were stained in the dark with LysoTracker Red DND-99 (50 nM, 1 ml, Thermo Fisher) or MitoTracker Red CMXRos working solution (100 nM, 1 ml, Thermo Fisher) at 37°C for 60 min. The chondrocytes were next fixed with 4% paraformaldehyde (4% PFA, Biosharp, China) for 15 min and stained the chondrocyte nuclei with DAPI (Beyotime). The chondrocytes were captured via a confocal microscopy (Leica, Germany) and the correlated Pearson’s *R* value was analyzed by Fiji software.

### Subcellular superoxide staining assay

The subcellular superoxide levels were detected by using a MitoSOX fluorescence probe (Yeason, China). Briefly, the treated chondrocytes (200 μM H_2_O_2_, 24 h) in six-well plates, with a density of 2 × 10^5^ cells/well, have been co-cultured with 20 μg/ml of UIO66-COOH, Mn_3_O_4_/UIO3 and Mn_3_O_4_/UIO-TPP for 24 h. Once washed with PBS buffer three times after discarding the medium, the cells were stained in the dark with 5 μM MitoSOX solution at 37°C for 10 min and then stained the chondrocyte nuclei with Hoechst 33342 (Solarbio). The chondrocytes were washed with PBS buffer three times before being captured via a fluorescence microscope and the mean fluorescence intensity (MFI) quantitative results were analyzed by Image J software.

### Intracellular mitochondrial membrane potential (ΔΨm) assay

Intracellular ΔΨm was measured by using a Mitochondrial membrane potential assay kit (Beyotime). Briefly, the treated chondrocytes (200 μM H_2_O_2_, 24 h) in 24-well plates, with a density of 4 × 10^4^ cells/well, have been co-cultured with 20 μg/ml of UIO66-COOH, Mn_3_O_4_/UIO3 and Mn_3_O_4_/UIO-TPP for 24 h. And were stained in the dark with 10 μM TMRE working solution at 37°C for 30 min, and then stained the chondrocyte nuclei with Hoechst 33342. The chondrocytes were washed with PBS buffer three times before being captured via a fluorescence microscope and the MFI quantitative results were analyzed by Image J software.

### Intracellular mitochondrial calcium level assay

Intracellular mitochondrial calcium level was measured by using Rhod2/AM fluorescent probe (Yeason, China). Briefly, the treated chondrocytes (200 μM H_2_O_2_, 24 h) in 24-well plates, with a density of 4 × 10^4^ cells/well, have been co-cultured with 20 μg/ml of UIO66-COOH, Mn_3_O_4_/UIO3 and Mn_3_O_4_/UIO-TPP for 24 h. And were stained in the dark with 10 μM Rhod2/AM working solution (containing 0.02% Pluronic F-127) at 37°C for 30 min, and then incubated with fresh DMEM for another 30 min. The chondrocytes were washed with PBS buffer three times before being captured via a fluorescence microscope and the MFI quantitative results were analyzed by Image J software.

### Intracellular ATP level assay

Intracellular ATP level was measured by using an ATP assay kit (Beyotime) based on the manufacturer’s guidelines. Briefly, the treated chondrocytes (200 μM H_2_O_2_, 24 h) in six-well plates, with a density of 2 × 10^5^ cells/well, have been co-cultured with 20 μg/ml of UIO66-COOH, Mn_3_O_4_/UIO3 and Mn_3_O_4_/UIO-TPP for 24 h. Once washed with PBS buffer three times after discarding the medium, the cells were collected and lysed in RIPA buffer. A volume of 20 μl supernatant was next isolated by centrifugation and was added to 100 μl ATP detection working solution in a 96-well plate, respectively, and the results were obtained via a fluorometer (BioTek, USA).

### Intracellular mtDNA evaluation

The intracellular mtDNA of the treated chondrocytes was collected using a Universal DNA extraction kit (Magen, China) based on the manufacturer’s guidelines. The mtDNA (mitochondrially encoded Cytochrome C Oxidase II, MT-CO2) copy number was further calculated by quantitative real-time PCR (qRT-PCR) proceeded following the steps of 95°C for 10 min, 95°C for 15 s and 60°C for 60 s via a Detection System (Roche, Switzerland), and ACTB (β-actin) was selected as the reference gene. The primer sequences were listed in Supplementary Table S4.

### Intracellular ROS level evaluation

The intracellular ROS level was measured by using a 2′,7′-dichlorofluorescin diacetate fluorescent probe (DCFH, Solarbio) Briefly, the treated chondrocytes (200 μM H_2_O_2_, 24 h) in six-well plates, with a density of 2 × 10^5^ cells/well, have been co-cultured with 20 μg/ml of UIO66-COOH, Mn_3_O_4_/UIO3 and Mn_3_O_4_/UIO-TPP for 24 h. Once washed with PBS buffer three times after discarding the medium, the cells were stained in the dark with 10 μM DCFH working solution at 37°C for 30 min. The chondrocytes were washed with PBS buffer three times before being captured via a fluorescence microscope and the MFI quantitative results were analyzed by Image J software.

### qRT-PCR analysis

The total RNA of the chondrocytes in experiments was obtained by using a Total RNA extraction kit (Magen, China) based on the manufacturer’s guidelines. After the obtained RNA was reverse transcript, the qRT-PCR was proceeded following the steps of 95°C for 10 min, 95°C for 15 s and 60°C for 60 s via a Detection System (Roche, Switzerland). The IL6, MMP13, COX2 and MMP3 gene expression levels were analyzed, and glyceraldehyde-3-phosphate dehydrogenase (GAPDH) was selected as the reference gene. The primary sequences were listed in Supplementary Table S4.

### Immunofluorescence staining

The IL6 and MMP13 protein expression levels were further analyzed by using immunofluorescence staining. Briefly, the treated chondrocytes (200 μM H_2_O_2_, 24 h) in six-well plates, with a density of 2 × 10^5^ cells/well, have been co-cultured with 20 μg/ml of UIO66-COOH, Mn_3_O_4_/UIO3 and Mn_3_O_4_/UIO-TPP for 24 h. Once the chondrocytes were fixed with 4% PFA for 20 min, 3% H_2_O_2_ was used for another 15 min before being blocked with goat serum working solution (ZS-bio, China) to eliminate nonspecific staining. Primary antibodies of IL-6 and MMP-13 (1:200, Bioss, China) were used to incubate with the chondrocytes overnight in a cool condition (4°C). Subsequently, an FITC-coupled anti-rabbit secondary antibody (1:100, Boster, China) was next used in the dark for 1 h, and the chondrocyte nuclei were stained with DAPI. The chondrocytes were further washed with PBS buffer three times before being captured via a fluorescence microscope and the MFI quantitative results were analyzed by Image J software. Besides, the evaluation of the DNA damage proceeded with the same treatments as described above, and the DNA damage of cells was measured based on the manufacturer’s guidelines by using a DNA damage assay kit by γ-H2AX immunofluorescence (Beyotime). The chondrocytes were further washed with PBS buffer three times before being captured via a fluorescence microscope and the MFI quantitative results were analyzed by Image J software.

### *In vivo* experiments

The animal experiment was sanctioned by the Animal Ethics Committee of Guangxi Medical University (No. 202106011). Male SD rats (140–180 g) were acquired from Guangxi Medical University Laboratory Animal Center, and the experimental OA animal models were next constructed by performing an anterior cruciate ligament transection (ACLT) surgery on these rats. After the surgery, the rats were randomly categorized into five groups: (i) Sham group: Normal rats have only incised the skin and joint capsule without any further process; (ii) OA group: OA rats with PBS buffer injection at the dosage of 100 μl, two times a week; (iii) UIO66-COOH group: OA rats with UIO66-COOH injection at the dosage of 100 μl (20 μg/ml), two times a week; (iv) Mn_3_O_4_/UIO3 group: OA rats with Mn_3_O_4_/UIO3 injection at the dosage of 100 μl (20 μg/ml), two times a week; (v) Mn_3_O_4_/UIO-TPP: OA rats with Mn_3_O_4_/UIO-TPP injection at the dosage of 100 μl (20 μg/ml), two times a week. An overdose of pentobarbital sodium (Nembutal) was carried out for the euthanasia of rats at 4 and 8 weeks, and the knee joints and other major organs of these rats were harvested for further experiments, the heart, liver, spleen, lung and kidney were included.

The collected knee joints of rats were partially captured and scored based on Pelletier’s macroscopic scores by three individuals, and the others were fixed for 2 days by using 4% PFA and decalcified for 6 weeks via an Ultrasonic Decalcifying Unit (USE 33, Germany) at pH = 7.2 with 0.5 M EDTA (Macklin). After that, 3 μm thick sections of each group were acquired after the decalcified knee joints were embedded and sliced. These sections were soon stained by using hematoxylin–eosin (HE, Solarbio) and safranin O-fast green (S-G, Solarbio), then captured via an optical microscope (Olympus BX53, Japan). The captured photographs were later scored based on the Mankin scores by three individuals. Meanwhile, the collected organs of these rats were also fixed for 2 days by using 4% PFA, then 3 μm thick organ sections of each group were acquired after the organs were embedded and sliced. And *in vivo* cytotoxicity was further investigated based on these organs’ sections by histological analysis.

To further investigated the inflammatory factors (IL-6 and MMP-13) expression levels in joint tissues. The synovial fluid harvested from the treated rats’ joints was also detected by using an enzyme-linked immunosorbent assay (ELISA, Meimian, China) based on the manufacturer’s guidelines. The results were obtained via a microplate reader at the absorbance of 450 nm.

To further investigate the retention time of Mn_3_O_4_/UIO-TPP nanozyme in the articular cavity of SD rats. Cy5.5-labeled Mn_3_O_4_/UIO-TPP nanozyme was firstly dispersed in PBS buffer to form a solution at the concentration of 20 μg/ml and was next injected 100 μl into the knee joints of rats. The fluorescence signals and fluorescence intensity quantification were recorded via *in vivo* imaging system (AniView 100, BLT, China) at various time points (0, 3, 6, 12, 24, 48, 72, 96 h). Different *ex vivo* organs were also harvested after 96 h, and then recorded and quantified fluorescence intensity via the same equipment.

### Statistical analysis

Statistical analysis of all the data obtained from the experiments was proceeded using SPSS 26.0 statistical software with at least three repeated experiments and reported as means ± standard deviations. The comparison between the two groups was proceeded by using one-way ANOVA with the least significant difference analysis. *P* < 0.05 was regarded statistically significant, and ^*^P<0.05, ^**^P<0.01, ^***^P<0.001 and ^****^P<0.0001.

## Results and discussion

### Synthesis and characterization of the Mn_3_O_4_/UIO nanozymes

**Figure 2.**
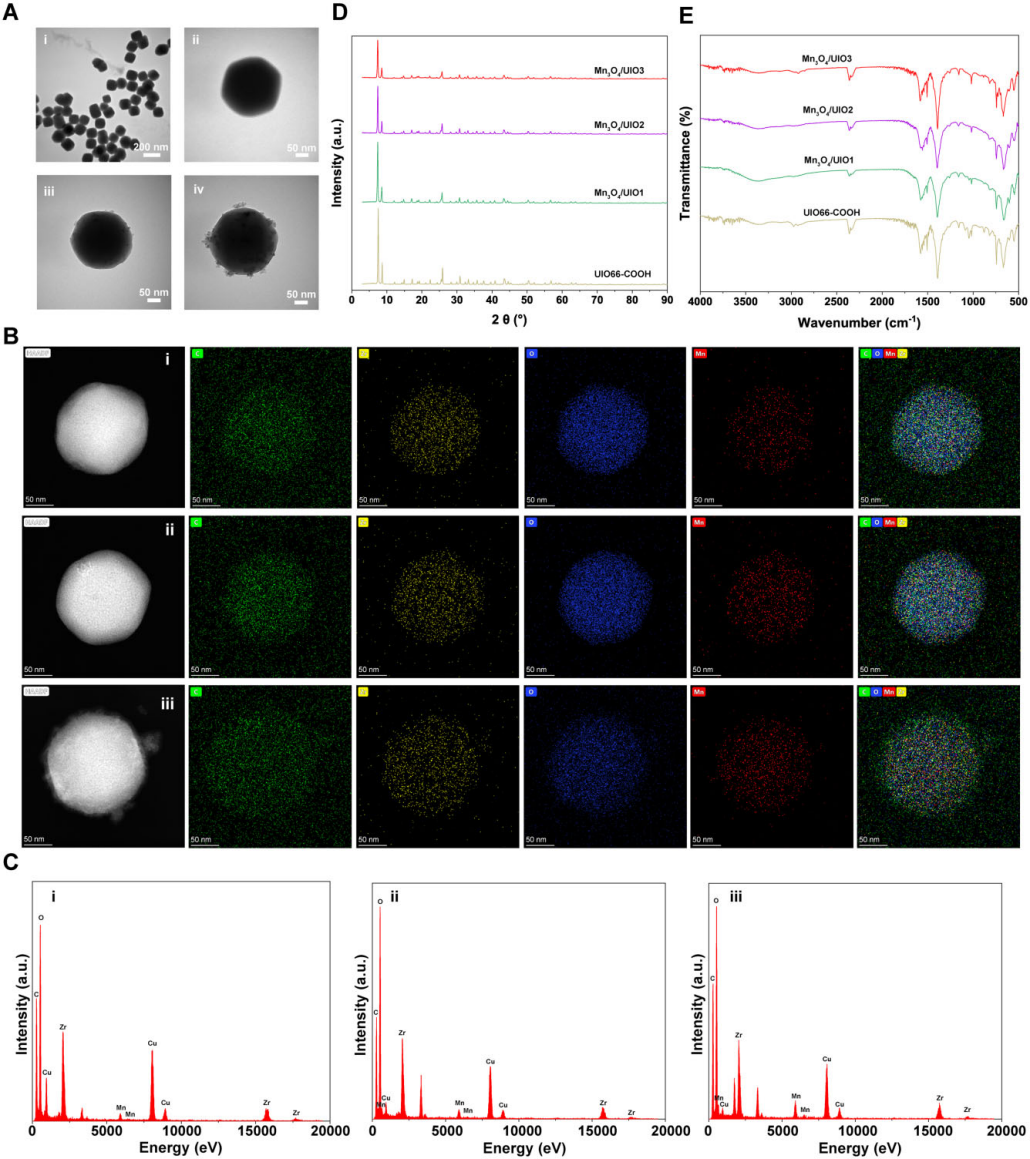
Characterization of Mn_3_O_4_/UIO nanozymes. (**A**) TEM images of UIO66-COOH (i), Mn_3_O_4_/UIO1 (ii), Mn_3_O_4_/UIO2 (iii) and Mn_3_O_4_/UIO3 (iv). (**B**) STEM and EDX mapping images of Mn_3_O_4_/UIO1 (i), Mn_3_O_4_/UIO2 (ii) and Mn_3_O_4_/UIO3 (iii). (HAADF: white, C: green, Zr: yellow, O: blue and Mn: red). (**C**) EDX element analysis of Mn_3_O_4_//UIO1 (i), Mn_3_O_4_/UIO2 (ii) and Mn_3_O_4_/UIO3 (iii). (**D**) XRD spectra of UIO66-COOH, Mn_3_O_4_//UIO1, Mn_3_O_4_/UIO2 and Mn_3_O_4_/UIO3. (**E**) FTIR spectra of UIO66-COOH, Mn_3_O_4_//UIO1, Mn_3_O_4_/UIO2 and Mn_3_O_4_/UIO3.

The Mn_3_O_4_/UIO nanozymes were synthesized based on the precursor UIO66-COOH loaded with different contents of Mn_3_O_4_. In brief, the UIO66-COOH NPs were synthesized and then redispersed in ethanol at 120°C with different amounts of Mn(CH_3_COO)_2_·4H_2_O (4, 16 and 32 mg, respectively) to form Mn_3_O_4_/UIOs (named Mn_3_O_4_/UIO1, Mn_3_O_4_/UIO2 and Mn_3_O_4_/UIO3) by electrostatic interaction [41]. TEM images (Figure 2A; Supplementary Figure S1) showed that compared with UIO66 and UIO66-COOH, Mn_3_O_4_/UIOs preserved its 3D octahedral structure and uniform size after Mn_3_O_4_ loading at the condition of Mn(CH_3_COO)_2_·4H_2_O as an Mn^2+^ source. An appropriate loading capacity was achieved while further increasing the quantity of Mn(CH_3_COO)_2_·4H_2_O to 64 mg resulting in unbound particles surrounding the Mn_3_O_4_/UIO (Supplementary Figure S2). DLS studies confirmed that compared with UIO66-COOH, the hydrodynamic diameters of Mn_3_O_4_/UIOs (Supplementary Figure S3) were slightly increased according to the amount of Mn^2+^. And the same trend was also shown for zeta potential with the order of Mn_3_O_4_/UIO1 < Mn_3_O_4_/UIO2 < Mn_3_O_4_/UIO3 (Supplementary Figure S4). Notably, the hydrodynamic diameters of the three Mn_3_O_4_/UIO nanozymes were far less than bare Mn_3_O_4_ NPs, indicating the favorable dispersibility in aqueous solution. EDX mapping results of Mn_3_O_4_/UIO further proved the existence of the Mn element in the NPs (Figure 2B and C).

XRD spectra (Figure 2D) of the synthesized UIO66-COOH illustrated iconic diffraction peaks at 2θ angles of 7.56°, 8.70°, 25.90°, 30.88°, 43.48°, 50.50° and 56.82°, almost consistent with standard UIO66 sample (CCDC No. 837796, Supplementary Figure S5A), indicating that the carboxylated process did not change its crystal structures. However, no iconic diffraction peaks of Mn_3_O_4_ (JCPDS24-0734, Supplementary Figure S5B) were detected in the patterns of Mn_3_O_4_/UIOs, probably due to that Mn_3_O_4_ crystals loaded on the UIO66-COOH were ultrasmall and hard to detect. Peaks at 1716.6 and 1734.2/cm of FTIR spectra (Figure 2E) and those at 1615/cm of Raman spectra (Supplementary Figure S6) verified free carboxylic acid and its dimer in both Mn_3_O_4_/UIOs and UIO66-COOH [42–44]. Yet, characteristics of FTIR or Raman spectra of Mn_3_O_4_ were hardly found in Mn_3_O_4_/UIOs because of weak signals from ultrasmall Mn_3_O_4_ crystals (Figure 2E; Supplementary Figures S6 and S7).

XPS results (Supplementary Figure S8A) further proved the apparent Mn element in the Mn_3_O_4_/UIOs. And the fine spectra of Mn 2p (Supplementary Figure S8B) showed the peaks of Mn 2p_3/2_ and Mn 2p_1/2_ at 641.1 and at 653.1 eV, respectively, indicating the Mn element was mainly presented as Mn_3_O_4_ in these samples. And the ratio of Mn^2+^ and Mn^3+^ ions in the samples was ∼1:2 according to the area ratio, which was consistent with the theoretical value reported previously [45–47]. Meanwhile, TGA results (Supplementary Figure S9) showed that compared with UIO66-COOH, Mn_3_O_4_/UIOs differ in the remaining mass percentage at 800°C, which was caused by the different content of Mn_3_O_4_ loading. And similar to the work previously reported [48], this increase of Mn_3_O_4_ loadings occupied the pore structure of MOFs also leading to a diminution in their BET surface area and pore volume (Supplementary Table S2). Further ICP-OES study (Supplementary Table S3) demonstrated that the mass fraction of Mn increased with the trend of Mn_3_O_4_/UIO1 < Mn_3_O_4_/UIO2 < Mn_3_O_4_/UIO3, were 0.80%, 3.39% and 5.16%, respectively.

The above all demonstrated the successful synthesis of Mn_3_O_4_/UIOs with varying levels of Mn_3_O_4_ loading. The loading of Mn_3_O_4_ did not significantly alter the crystal and molecular structure of UIO66-COOH. But the morphology structure, element ratios, zeta potential and BET-specific surface area were changed after loading.

### Antioxidant enzyme-like activity

SOD enzyme catalyzed ·$O_{2}^{-}$ into H_2_O_2_ and then CAT enzyme decomposes H_2_O_2_ into innoxious O_2_ and H_2_O, which was a typical ROS clearance route *in vivo*.

**Figure 3.**
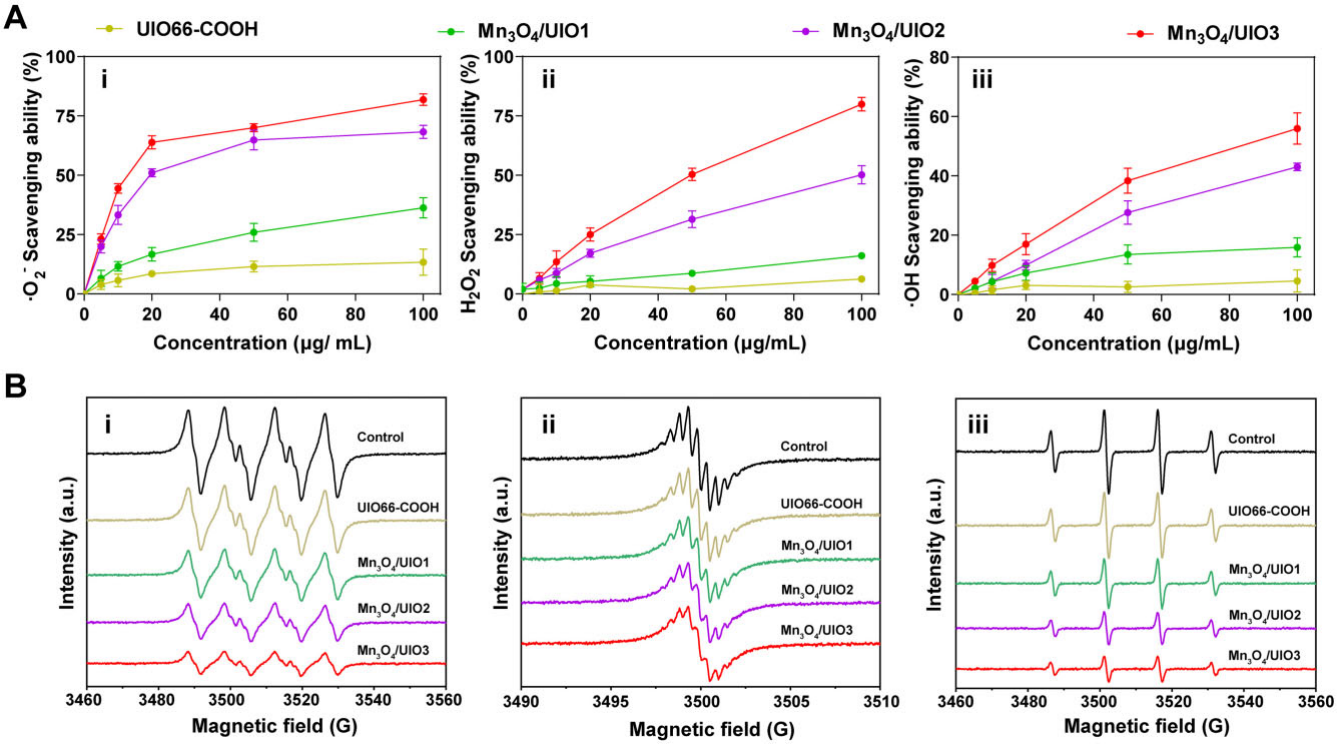
ROS Scavenging capacity of Mn_3_O_4_/UIOs. (**A**) ROS scavenging ability of UIO66-COOH, Mn_3_O_4_/UIO1, Mn_3_O_4_/UIO2 and Mn_3_O_4_/UIO3 detected by ROS clearing kits at multiple concentrations (0, 5, 10, 20, 50, 100 μg/ml): ·$O_{2}^{-}$ (i), H_2_O_2_ (ii) and ·OH (iii). (**B**) ROS scavenging ability of UIO66-COOH, Mn_3_O_4_/UIO1, Mn_3_O_4_/UIO2 and Mn_3_O_4_/UIO3 assessed via ESR at a concentration of 20 μg/ml: ·$O_{2}^{-}$ (i), H_2_O_2_ (ii) and ·OH (iii).

Herein, the scavenging capability of ·$O_{2}^{-}$ was measured to evaluate the SOD-like activity of Mn_3_O_4_/UIOs. As shown in (i) of Figure 3A, all Mn_3_O_4_/UIOs showed a concentration-dependent manner for ·$O_{2}^{-}$ scavenging, while UIO66-COOH has negligible ·$O_{2}^{-}$ scavenging capacity. Noticeably, ∼81.9% of ·$O_{2}^{-}$ could be eliminated by Mn_3_O_4_/UIO3, much higher than Mn_3_O_4_/UIO1 and Mn_3_O_4_/UIO2.

H_2_O_2_ as a secondary product of ·$O_{2}^{-}$ was evaluated to test the CAT-like activity of Mn_3_O_4_/UIOs later. As results, UIO66-COOH had no obvious scavenging capability for H_2_O_2._ In contrast, Mn_3_O_4_/UIOs produced effect on H_2_O_2_ scavenging the order of Mn_3_O_4_/UIO1 < Mn_3_O_4_/UIO2 < Mn_3_O_4_/UIO3 (Figure 3A, ii), especially for Mn_3_O_4_/UIO3 achieved a conspicuous H_2_O_2_ clearance ∼80.0%.

In addition, ·OH as another kind of important ROS which could do harm to cells and tissues seriously was further investigated. The results illustrated UIO66-COOH failed to eliminate ·OH, but Mn_3_O_4_/UIO3 exhibited remarkable ·OH scavenging activity with the trend of Mn_3_O_4_/UIO1 < Mn_3_O_4_/UIO2 < Mn_3_O_4_/UIO3. Particularly, Mn_3_O_4_/UIO3 showed 56.0% ·OH scavenging (Figure 3A, iii).

Further, ESR results (Figure 3B) proved that Mn_3_O_4_/UIOs, particularly Mn_3_O_4_/UIO3, could distinctly decrease the signals of ·$O_{2}^{-}$, H_2_O_2_ and ·OH compared with control and UIO66-COOH, confirming the superior SOD-like, CAT-like and ·OH scavenging activities.

Based on the above results, it demonstrated a sequential catalysis behavior of Mn3O4/UIOs for ROS scavenging, especially for Mn_3_O_4_/UIO3 due to its highest absolute content of Mn_3_O_4_, potentiating the application for OA therapy.

### Cell viability

**Figure 4.**
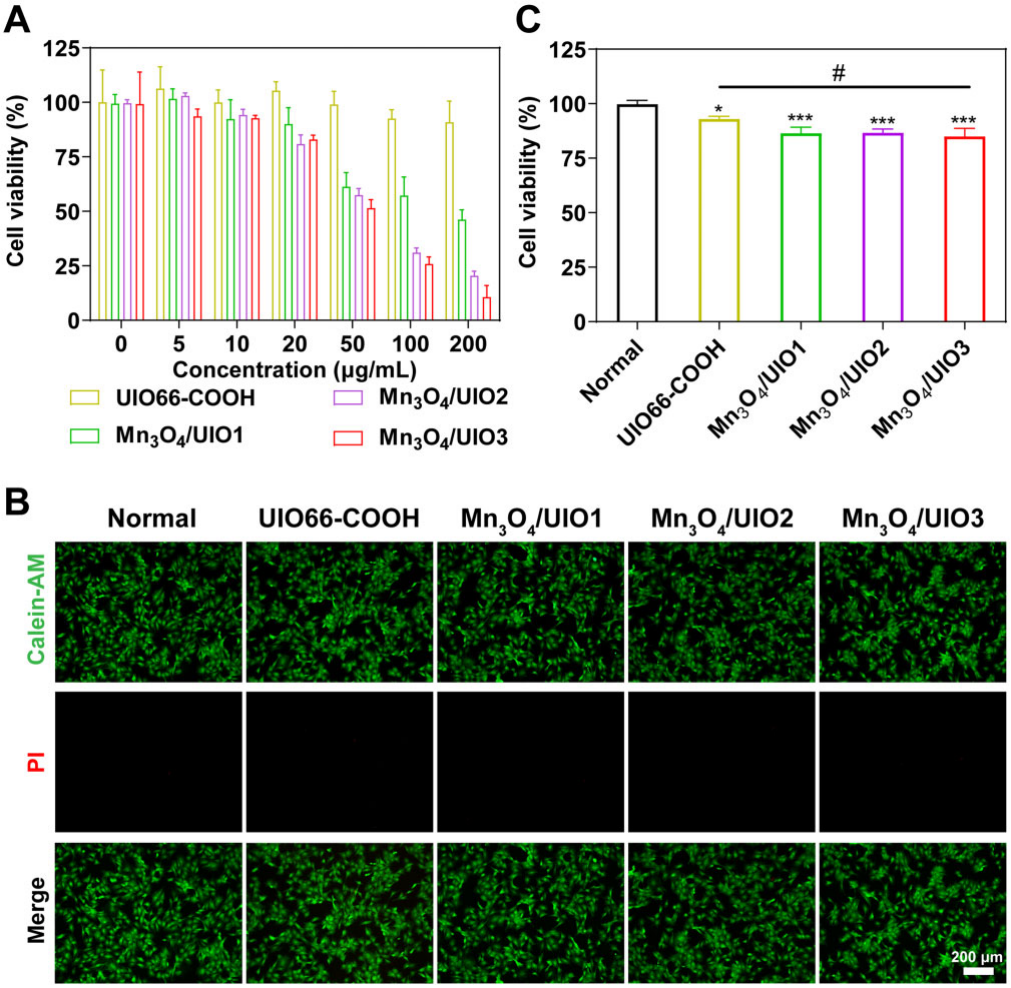
Biocompatibility *in vitro*. (**A**) Cytotoxicity by using CCK-8 assay of UIO66-COOH, Mn_3_O_4_/UIO1, Mn_3_O_4_/UIO2 and Mn_3_O_4_/UIO3. (**B**) Live and dead staining results after incubating with UIO66-COOH, Mn_3_O_4_/UIO1, Mn_3_O_4_/UIO2 and Mn_3_O_4_/UIO3 (20 μg/ml), and the correlated quantitative results (**C**). ‘*’ denotes a comparison with the normal group, *P < 0.05, **P < 0.01 and ***P < 0.001, and ‘#’ denotes a comparison between two groups, ^#^*P* < 0.05.

The cytotoxicity of Mn_3_O_4_/UIOs were detected by using a CCK-8 assay kit (Figure 4A). As shown, UIO66-COOH displayed no obvious cytotoxicity at all concentration sets. Meanwhile, Mn_3_O_4_/UIO nanozymes only showed little cytotoxicity at a concentration of 5–20 μg/ml with cell viability maintained at no <80%. Once the concentration of Mn_3_O_4_/UIOs increased upper than 50 μg/ml, the cell viability would decrease to lower than 70%. For comparison, bare Mn_3_O_4_ NPs showed cytotoxic effect at the concentration of 5 μg/ml in Supplementary Figure S10. This biocompatibility difference was probably due to the released Mn ions into the cytoplasm after endocytosis of the material, because free Mn ions from ultra-small manganese oxides were reported to have cytotoxicity [34]. Therefore, without apparent cytotoxicity, 20 μg/ml Mn_3_O_4_/UIO nanozymes were chosen for further experiments (Figure 4B and 4C). Meanwhile, live and dead staining of chondrocytes also confirmed this. Considering the amalgamation of catalytic proficiency and biocompatibility, a concentration of 20 μg/ml was preferred in the subsequent experiments of Mn_3_O_4_/UIO3 nanozyme.

### Conjugation with mitochondria-targeting TPP group to form Mn_3_O_4_/UIO-TPP

The existence of an oxidative respiratory pathway and ion pump in mitochondria maintains its membrane potential difference of ∼180 mV [49], making it highly affinity for some chemical molecules by charge attraction, such as the TPP group [50]. TPP group was combined with the free carboxyl group on the surface of UIO66-COOH via amidation through a classical NHS/EDC reaction system to form a mitochondrial-targeting Mn_3_O_4_/UIO-TPP nanozyme.

The FTIR spectra of the prepared Mn_3_O_4_/UIO-TPP nanozyme were compared with those of Mn_3_O_4_/UIO3 (Supplementary Figure S11), and the broad peak at the wavenumber of 2500–3200/cm, corresponded to free carboxylic acid O–H bonds, were decreased, which were caused by the consumption of carboxyl groups to form amide bonds. Correspondingly, Mn_3_O_4_/UIO-TPP showed a new absorption peak at wavenumber of 1660–1725/cm, correlated to C=O bonds Band I in amide bonds [51]. Besides, the surface charge of Mn_3_O_4_/UIO-TPP turned to 8.01 mV (Supplementary Figure S12A), with little significant changes in its hydrodynamic diameter (Supplementary Figure S12B). All above illustrated the successful fabrication of Mn_3_O_4_/UIO-TPP nanozyme.

### Subcellular tracking and mitochondrial-targeting

Mitochondria were the primary generator of intracellular superoxide and the origin of cellular oxidative damage [52, 53], thus the subcellular location of the nanozymes was critical for the scavenging of subcellular ROS. However, previous reported NPs as antioxidant drugs for mitochondria ROS scavenging were unrealistic in targeting effect by systemic administration [54], turned out to a weak ROS scavenging efficiency, and were easily cleared and metabolized in a short period [55], consequently resulting in limited therapeutic effect. This led to the need for repeated dosing and further increasing the toxic risk of the treatment, which also revealed improvements were still needed in the research of the relevant field.

**Figure 5.**
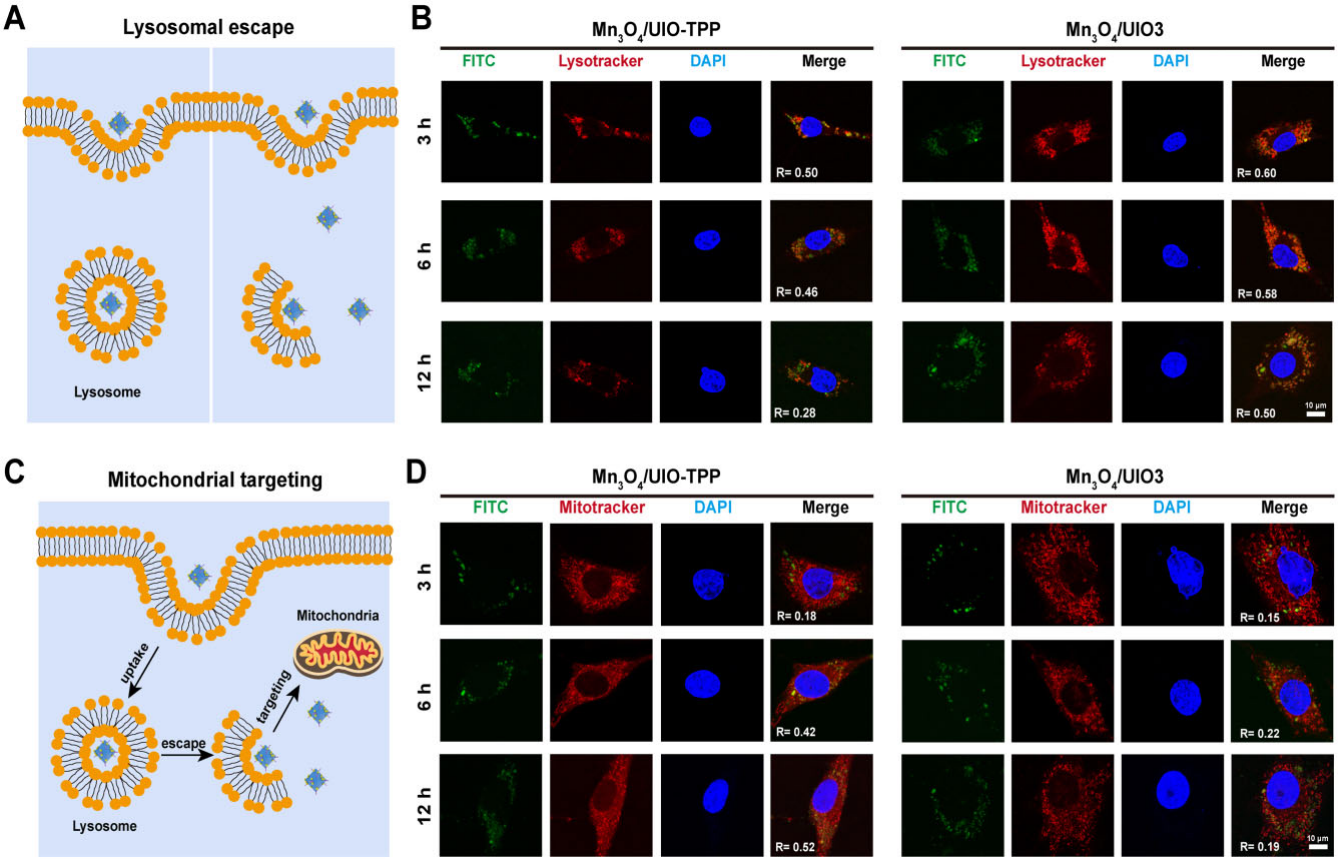
Lysosomal escape and mitochondrial-targeting of Mn_3_O_4_/UIO-TPP nanozyme. (**A**) Scheme of NPs intracellular localization uptake by chondrocyte. (**B**) Confocal images of lysosome escape of Mn_3_O_4_/UIO-TPP and Mn_3_O_4_/UIO3 after incubated with chondrocytes for 3, 6 and 12 h, respectively. (FITC-labeled Mn_3_O_4_/UIO3 and Mn_3_O_4_/UIO-TPP: green. Lysosomes stained with lysotracker red: red. Chondrocyte nuclei stained with DAPI: blue). (**C**) Schematic of Mn_3_O_4_/UIO-TPP intracellular moving route. (**D**) Confocal images of the colocalized Mn_3_O_4_/UIO-TPP and Mn_3_O_4_/UIO3 with mitochondria after lysosomal escape for 3, 6 and 12 h, respectively (FITC-labeled Mn_3_O_4_/UIO3 and Mn_3_O_4_/UIO-TPP: green. Mitochondria stained with mitotracker red: red. Nuclei stained with DAPI: blue. Overlap *R* means the overlap ratio determined by Fiji software).

Generally, the nanomaterials were first transported into lysosomes after cellular uptake [56], which may prevent effective targeting of NPs to mitochondria. It is imperative to break the lysosomal barriers to improve ROS scavenging efficiency [57]. In our study, following the cellular uptake in chondrocytes, the nanozymes were located in lysosomes (Figure 5A). After incubation for 3 h, both FITC-labeled Mn_3_O_4_/UIO3 and Mn_3_O_4_/UIO-TPP showed strong colocalization signals primarily with lysosomes stained by LysoTracker Red (*R* = 0.60 and *R* = 0.50, Figure 5B). After 12 h, the colocalization coefficient with lysosomes of Mn_3_O_4_/UIO-TPP treated cells was significantly reduced, while it slightly decreased for Mn_3_O_4_/UIO3-treated cells, demonstrating the accelerated lysosomal escape effect of Mn_3_O_4_/UIO-TPP achieved by the positive surface charge after the TPP conjugation.

The capability of Mn_3_O_4_/UIO-TPP of mitochondrial-targeting (Figure 5C) was further investigated. Following labeling the mitochondria with MitoTracker Red, it was obvious to found Mn_3_O_4_/UIO-TPP showed preferential mitochondrial accumulation. After 12 h incubation, colocalization analysis (Figure 5D) illustrated the colocalization coefficient with mitochondria of Mn_3_O_4_/UIO-TPP nanozyme was *R* = 0.52, higher than that of Mn_3_O_4_/UIO3 (*R* = 0.19), revealing that Mn_3_O_4_/UIO-TPP nanozyme have the ability of breaking lysosomal barriers and mitochondrial-targeting.

### Inhibition of mitochondrial damage

**Figure 6.**
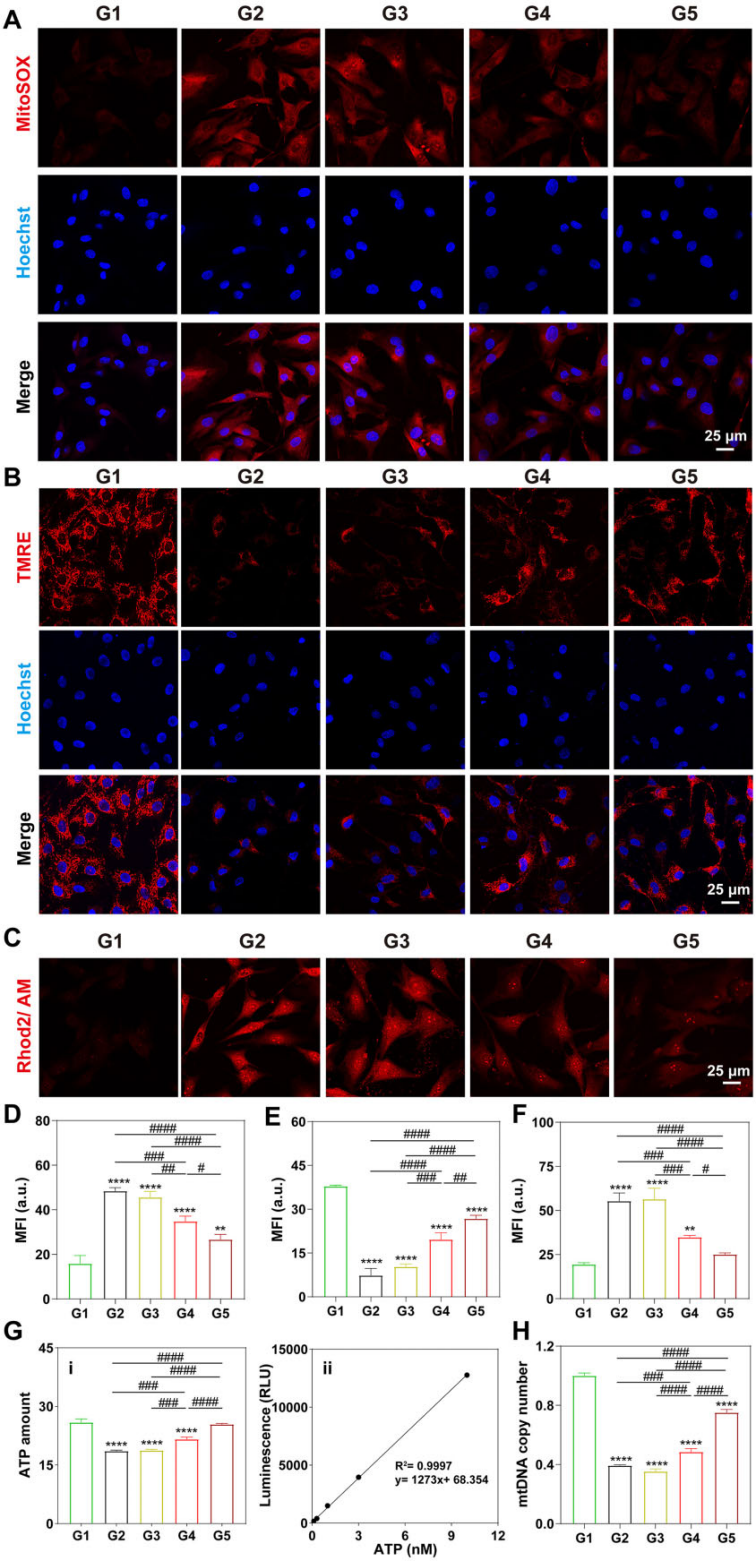
Mitochondrial protective effect of Mn_3_O_4_/UIO-TPP nanozyme. (**A**) Mitochondrial superoxide staining with different treatments by using a MitoSOX probe and the correlated fluorescence quantitative results (**D**). (**B**) ΔΨm levels by using a TMRE probe and the correlated fluorescence quantitative results (**E**). (**C**) Mitochondrial calcium levels by using Rhod2/AM probe as an indicator and the correlated fluorescence quantitative results (**F**). (**G**) Effect of Mn_3_O_4_/UIO-TPP nanozyme on intracellular ATP levels (i) and the correlated standard curve (ii). (**H**) Effect of Mn_3_O_4_/UIO-TPP nanozyme on mtDNA copy number by qRT-PCR (G1: Normal, G2: H_2_O_2_, G3: H_2_O_2_ + UIO66-COOH, G4: H_2_O_2_ + Mn_3_O_4_/UIO3, G5: H_2_O_2_ + Mn_3_O_4_/UIO-TPP. H_2_O_2_ concentration: 200 μM. ‘*’ denotes a comparison with normal group, *P < 0.05, **P < 0.01, ***P < 0.001 and ****P < 0.0001, and ‘#’ denotes a comparison between two groups, ^#^*P* < 0.05, ^##^*P* < 0.01, ^###^*P* < 0.001 and ^####^*P* < 0.0001.

In order to reveal the intracellular functions of Mn_3_O_4_/UIO-TPP nanozyme on mitochondria under oxidative stress conditions, H_2_O_2_ was used to induce chondrocytes to a state of oxidative stress, and mitochondrial ROS levels were first detected by MitoSOX fluorescence probe. As shown in Figure 6A and D, chondrocytes (G2) emitted a strong red fluorescent signal after being treated with H_2_O_2_ or H_2_O_2_ + UIO66-COOH compared with the normal group (G1), which was lowered after treatment by Mn_3_O_4_/UIO3 (G4) or Mn_3_O_4_/UIO-TPP (G5) nanozyme. Besides, quantification analysis indicated that Mn_3_O_4_/UIO-TPP nanozyme (G5) were more efficient in mitochondrial superoxide scavenging than Mn_3_O_4_/UIO3 (G4), which means that Mn_3_O_4_/UIO-TPP nanozyme have a better mitochondrial protective function.

This advantage of mitochondrial protective function was also manifested in promoting ΔΨm recovery. The maintenance of ΔΨm is crucial for the proper functioning of mitochondria, as it enables electron transfer through an intact electron transport chain and facilitates ATP generation, among other essential mitochondrial activities [49]. However, ΔΨm was fragile and easy to depolarize under oxidative stress conditions [58]. Herein, the protective effect of Mn_3_O_4_/UIO-TPP nanozyme on oxidative stress-induced ΔΨm depolarization was evaluated by using a tetramethylrhodamine ethyl ester perchlorate (TMRE) fluorescent probe. Normally, the TMRE probe accumulated rapidly in healthy mitochondrial and generated a red fluorescence. When cells were under oxidative stress conditions, negligible fluorescence was detected compared with the normal group (G1). However, after incubation with additional Mn_3_O_4_/UIO3 or Mn_3_O_4_/UIO-TPP nanozyme, an apparent increase in the MFI could be detected (Figure 6B). The quantitative results (Figure 6E) showed that the Mn_3_O_4_/UIO-TPP group (G5) presented the highest fluorescence intensity among the experimental groups, restored to 70.8% of the normal group (G1), indicating Mn_3_O_4_/UIO-TPP could recover the ΔΨm levels of chondrocytes due to oxidative damage to a certain extent.

Moreover, excessive intracellular ROS would induce mitochondrial Ca^2+^ overload, unbalanced calcium homeostasis, mitochondrial function failure and cell death [59]. Thereby, a mitochondrial Ca^2+^-specific Rhod2/AM fluorescent probe was next used to assess the intracellular mitochondrial calcium levels. As shown in Figure 6C and F of Ca^2+^ levels, the fluorescence intensity increased within chondrocytes mitochondria after being stimulated with H_2_O_2_ (G2), which was particularly reversed by Mn_3_O_4_/UIO-TPP (G5) nanozyme with MFI decreased by 54.6% compared with H_2_O_2_ treated group (G2). The results indicated that Mn_3_O_4_/UIO-TPP nanozyme could rebalance mitochondrial calcium homeostasis under oxidative stress conditions. And due to intracellular ATP being mainly produced by mitochondria [60], ATP levels could also be used as a measure of mitochondrial function. As results in Figure 6G, Mn_3_O_4_/UIO-TPP nanozyme sufficiently alleviated the reduction of ATP in oxidative damaged cells.

Finally, mtDNA expression levels as another important criterion [61] were further performed to evaluate mitochondrial function by qRT-PCR via its copy number (Figure 6H). The increased protective effect of Mn_3_O_4_/UIO-TPP nanozyme on mtDNA was better than Mn_3_O_4_/UIO3, implying that Mn_3_O_4_/UIO-TPP nanozyme was sufficient to eliminate mitochondrial superoxide, providing efficient protection of mitochondrial functions.

### Inhibition of oxidative and inflammatory in H_2_O_2_-induced chondrocytes

To estimate the antioxidant and anti-inflammatory potency of Mn_3_O_4_/UIO-TPP in H_2_O_2_-induced chondrocytes, the survival of chondrocytes was first detected by live and dead staining. As shown in Supplementary Figure S13, H_2_O_2_ pro-treated chondrocytes had a cell viability of 35.1%, demonstrating oxidative stress injury. However, Mn_3_O_4_/UIO3 and Mn_3_O_4_/UIO-TPP increased the cell viability to 63.3% and 77.0%, indicating they could prevent the H_2_O_2_-induced death of cells.

**Figure 7.**
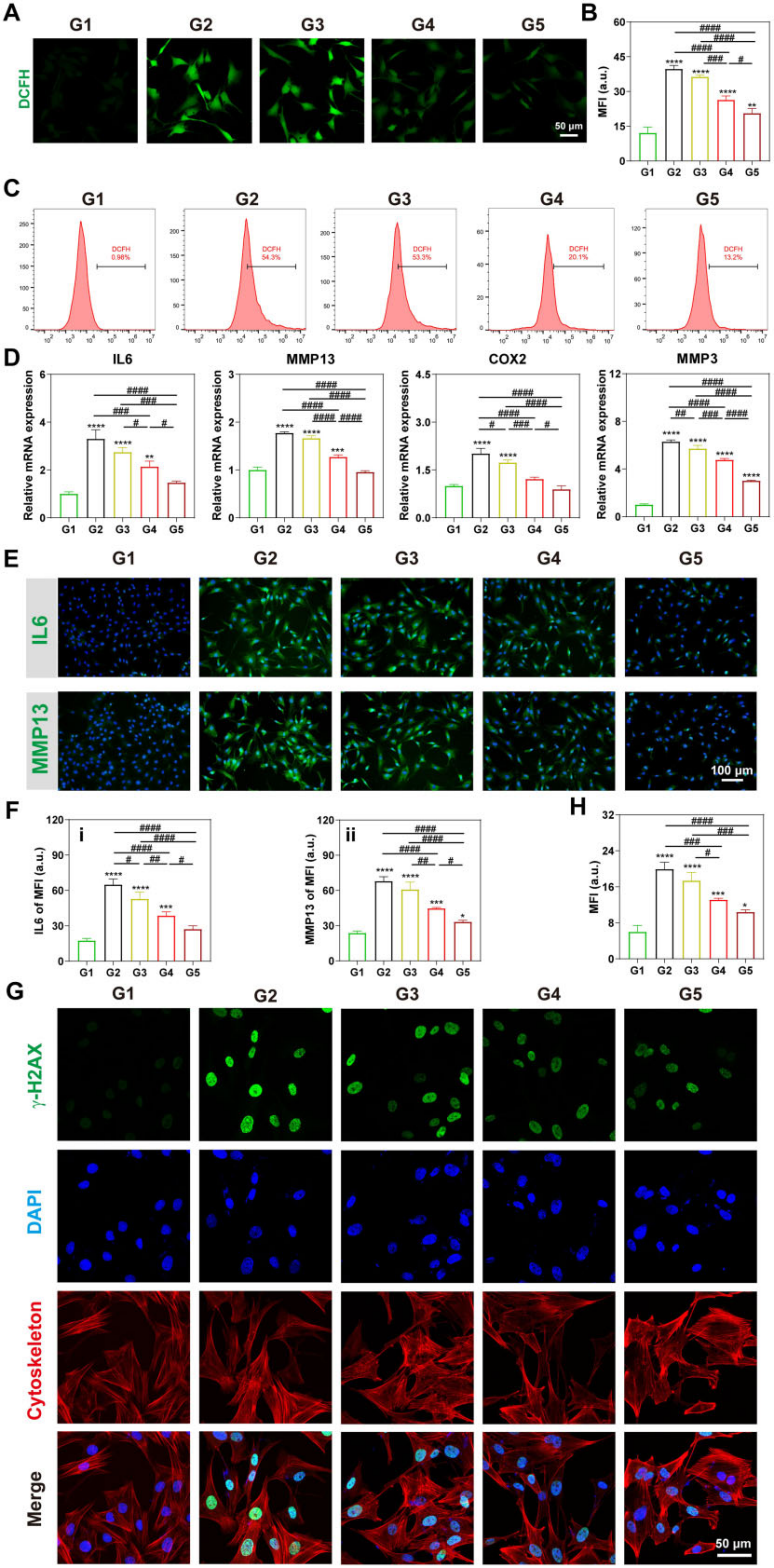
Antioxidant and anti-inflammatory effect of Mn_3_O_4_/UIO-TPP nanozyme. (**A**) Intracellular ROS staining by using a DCFH probe and the correlated fluorescence quantification results (**B**). (**C**) Intracellular ROS scavenging results via flow cytometry. (**D**) Expression levels of pro-inflammatory factors of IL6, MMP13, COX2 and MMP3 after different treatments by qRT-PCR. (**E**) Immunofluorescence staining of IL6 and MMP13 (FITC-labeled secondary antibody: green. Chondrocyte nuclei stained with DAPI: blue). (**F**) Fluorescence quantitative results of IL6 (i) and MMP13 (ii). (**G**) γ-H2AX foci immunofluorescence staining of chondrocytes under different treatments for evaluating DNA damage and the correlated fluorescence quantitative results (**H**). (FITC-labeled secondary antibody: green. Chondrocyte nuclei stained with DAPI: blue. Cytoskeleton: red). (G1: Normal, G2: H_2_O_2_, G3: H_2_O_2_ + UIO66-COOH, G4: H_2_O_2_ + Mn_3_O_4_/UIO3, G5: H_2_O_2_ + Mn_3_O_4_/UIO-TPP. H_2_O_2_ concentration: 200 μM. ‘*’ denotes a comparison with normal group, *P < 0.05, **P < 0.01, ***P < 0.001 and ****P < 0.0001, and ‘#’ denotes a comparison between two groups, ^#^*P* < 0.05, ^##^*P* < 0.01, ^###^*P* < 0.001 and ^####^*P* < 0.0001).

Later, the intracellular ROS levels were identified by a DCFH probe. Briefly, DCFH is an ROS probe that generated a green fluorescence when oxidized by intracellular ROS. Obviously, more intense green fluorescent signals could be noticed in Figure 7A and B for the H_2_O_2_-treated group (G2) compared with the normal group (G1). However, Mn_3_O_4_/UIO3 and Mn_3_O_4_/UIO-TPP significantly weakened fluorescence, especially for the Mn_3_O_4_/UIO-TPP group (G5), with the MFI reduction of 48.2% compared to the H_2_O_2_ group (G2). Moreover, the intracellular ROS levels of chondrocytes was also investigated via a flow cytometry after labeling with DCFH (Figure 7C), and the trend presented was consistent with that under the fluorescence microscope.

The anti-inflammatory ability of Mn_3_O_4_/UIO-TPP nanozyme was measured by qRT-PCR. The induction of H_2_O_2_ caused a significant upregulation of IL6, MMP13, COX2 and MMP3 expression levels (Figure 7D), which were pro-inflammatory factors and high correlation with OA inflammation [62–65]. Compared with the H_2_O_2_ group (G2), Mn_3_O_4_/UIO-TPP efficiently downregulated the gene expression of IL6, MMP13, COX2 and MMP3, which was also confirmed by immunofluorescence staining of IL6 and MMP13 (Figure 7E and F). Mn_3_O_4_/UIO-TPP nanozyme also significantly reduced the H_2_O_2_-induced oxidative damage of cellular DNA, which was demonstrated by the detection of γ-H2AX foci (Figure 6G and H).

All the above results indicated that Mn_3_O_4_/UIO-TPP nanozyme, as a multifunctional antioxidant, was effective in scavenging cellular ROS, anti-oxidative stress and anti-inflammatory in OA.

### *In vivo* curative effect for therapy of OA

**Figure 8.**
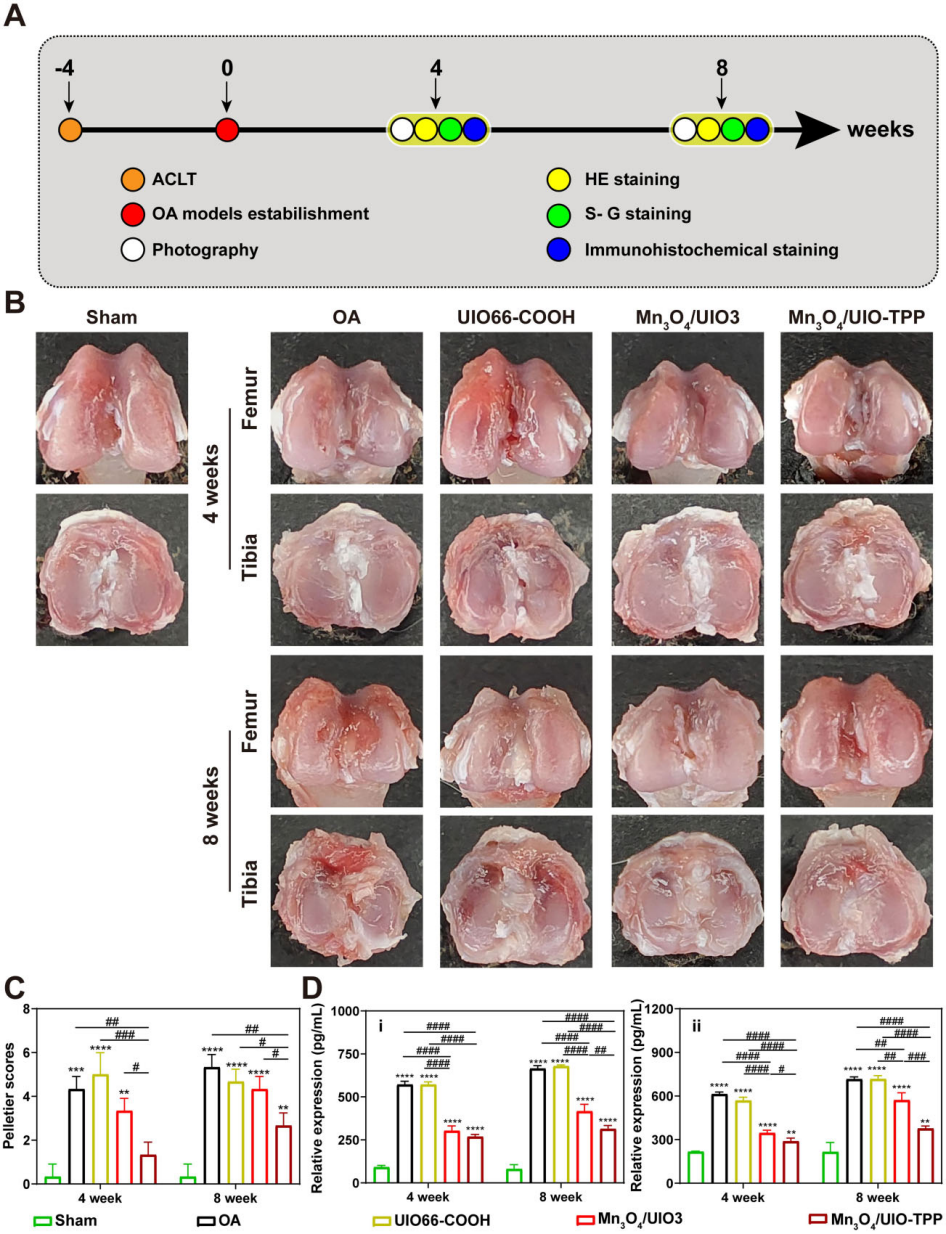
The therapeutic effects of Mn_3_O_4_/UIO-TPP nanozyme for OA. (**A**) Scheme of animal experiments. (**B**) Macroscopic images of SD rat OA knee joint cartilages collected at 4 and 8 weeks after IA injection, and the correlated Pelletier scores (**C**). (**D**) Relative inflammatory factors expression levels by ELISA (IL6 (i) and MMP13 (ii)). ‘*’ denotes a comparison with sham group, *P < 0.05, **P < 0.01, ***P < 0.001 and ****P < 0.0001, and ‘#’ denotes a comparison between two groups, ^#^*P* < 0.05, ^##^*P* < 0.01 , ^###^*P* < 0.001 and ^####^*P* < 0.0001.

In order to further estimate the *in vivo* curative effect of OA therapy, an ACLT surgery was performed to construct the OA models on SD rats. Once the OA models were constructed, an overdose of pentobarbital sodium (Nembutal) was carried out for the euthanasia of rats at 4 and 8 weeks, and the intra-articular (IA) injected knee joints were harvested after that (Figure 8A).

Macroscopic observation (Figure 8B) for the collected knee joints showed that the femoral plateau and tibial plateau were smooth and shiny in the sham group, however, both plateaus showed severe wear and damage over time in the OA group. More importantly, after IA injection of Mn_3_O_4_/UIO-TPP nanozyme, the degree of destruction of the femoral plateau and the tibial plateau was greatly reduced in the Mn_3_O_4_/UIO3 and Mn_3_O_4_/UIO-TPP groups compared with the OA group, especially for Mn_3_O_4_/UIO-TPP group. The results of Pelletier scores (Figure 8C) showed the OA group scored 4.33 and 5.33 at 4 and 8 weeks, respectively. However, the Mn_3_O_4_/UIO-TPP group was scored as 1.33 and 2.67, which decreased by 69.3% and 49.9% compared to the OA group, respectively. Besides, in order to investigate the levels of IL6 and MMP13 expression in the joint cavity, synovial fluid was extracted and analyzed using ELISA. The results of the Mn_3_O_4_/UIO-TPP group in Figure 8D and Supplementary Figure S14 showed a significant reduction in the expression of IL6 and MMP13 in chondrocytes, illustrating its excellent anti-inflammatory effect *in vivo*.

**Figure 9.**
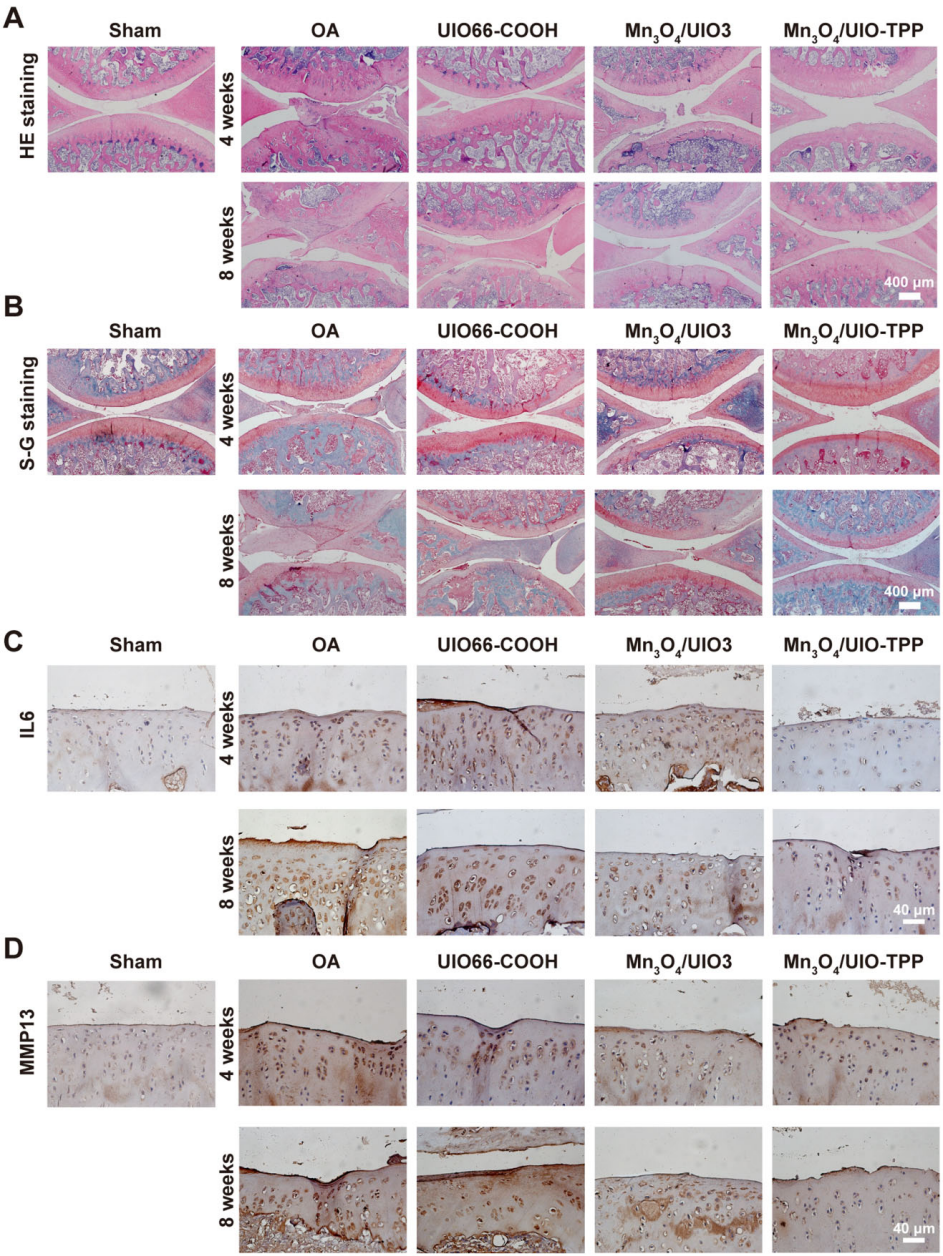
Morphological and pathological images after Mn_3_O_4_/UIO-TPP nanozyme treatment. (**A**) HE staining images. (**B**) S-G staining images. And immunohistochemical staining results of IL6 (**C**) and MMP13 (**D**).

Furthermore, histological staining further demonstrated the therapeutic effect of Mn_3_O_4_/UIO3 and Mn_3_O_4_/UIO-TPP. The HE staining results shown in Figure 9A illustrated chondrocyte loss and collagen disruption that occurred over time on the joint surface of the OA group. S–G staining (Figure 9B) further confirmed this, in the OA group, cartilage erosion and proteoglycan loss with faint staining of safranine O were apparent on the joint surface. Importantly, these changes were well improved after IA injection of Mn_3_O_4_/UIO-TPP. The staining results of the Mn_3_O_4_/UIO-TPP group showed a smooth cartilage surface, close to the normal chondrocytes, which was further supported by Mankin scores (Supplementary Figure S15). Moreover, inflammatory factors (IL6 and MMP13) expression levels in chondrocytes were also evaluated by using immunohistochemical staining. As shown in Figure 9C and D, there was positive staining (dark brown) on the articular surface of the OA group, indicating the highly expressed IL6 and MMP13, which were greatly decreased in the Mn_3_O_4_/UIO-TPP group.

Meanwhile, in order to evaluate the retention time of Mn_3_O_4_/UIO-TPP nanozyme *in vivo*, Cy5.5-labeled Mn_3_O_4_/UIO-TPP was detected via an *in vivo* imaging system after IA injection (Supplementary Figure S16A). Since a knee joint injection of Mn_3_O_4_/UIO-TPP nanozyme was performed, a slow decrease of fluorescence intensity was detected in the first 6 h, and the fluorescence signals persevere at a high level for another 48 h (Supplementary Figure S16B). Furthermore, these fluorescence signals were also presented in other organs of SD rats (Supplementary Figure S17), especially for the kidney, spleen and liver, indicating that Mn_3_O_4_/UIO-TPP nanozyme could be cleared by hepatic and renal pathways.

Major organs were harvested after IA injection for *in vivo* cytotoxicity evaluation, the heart, liver, spleen, lung and kidney were included. And the histological images of these organs of rats were shown in Supplementary Figure S18. All organs of Mn_3_O_4_/UIO-TPP treated rats presented no apparent damages, similar to the sham group.

All above has demonstrated that mitochondrial-targeting Mn_3_O_4_/UIO-TPP nanozyme has a realistic therapeutic effect on OA, with favorable biosafety, outstanding stability and long retention in the joint cavity.

## Conclusions

In summary, an artificially mitochondrial-targeting Mn_3_O_4_/UIO-TPP nanozyme as a potent ROS scavenger was developed by grafting Mn_3_O_4_ NPs with UIO66 series MOFs and mitochondria-targeting triphenylphosphine (TPP) groups for OA therapy. Results showed that Mn_3_O_4_/UIO-TPP nanozyme possesses efficient cascade catalysis for ·$O_{2}^{-}$ and H_2_O_2_, presenting SOD-like, CAT-like and ·OH scavenging activities.

By targeting mitochondria, Mn_3_O_4_/UIO-TPP nanozyme significantly normalized and rehabilitated the functions of ROS-induced damaged mitochondria and prevent chondrocyte apoptosis. Furthermore, Mn_3_O_4_/UIO-TPP nanozyme also decreased pro-inflammatory factors and reduced the DNA damage of chondrocytes without noticeable cytotoxicity. Mn_3_O_4_/UIO-TPP exhibited a realistic therapeutic effect on OA joints, with remarkable mitigation of OA progression and enhanced joint functions, it granting a wide application prospect in the therapy of OA and other ROS-related diseases.

## Supplementary Material

rbad078_Supplementary_Data
