# Supplementary material for: Mitochondrial-targeting Mn3O4/UIO-TPP nanozyme scavenge ROS to restore mitochondrial function for osteoarthritis therapy
Source: Regen Biomater. 2023 Sep 1;10:rbad078. doi: 10.1093/rb/rbad078 (PMC10640395; doi:10.1093/rb/rbad078)
Supplement: rbad078_Supplementary_Data [file rbad078_supplementary_data.docx]

**Supporting Information**

**Mitochondrial-targeting Mn_3_O_4_/UIO-TPP nanozyme scavenge ROS to restore mitochondrial function for osteoarthritis therapy**

Shengqing Zhang ^a, b #^, Jinhong Cai ^a, b #^, Yi Yao ^b, c #^, Lanli Huang ^b^, Li Zheng ^a, b *^ and Jinmin Zhao ^a, b, d *^


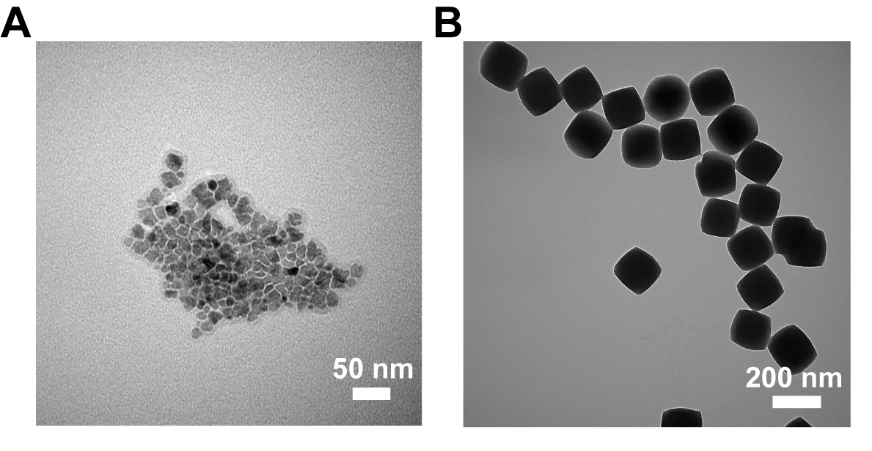


Figure S1. TEM images of Mn_3_O_4_ (A) and UIO66 (B).


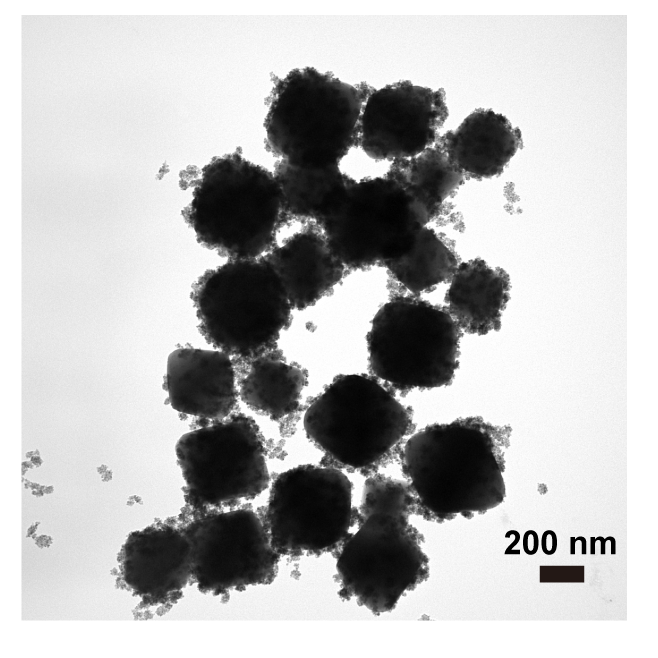


Figure S2. TEM images of Mn_3_O_4_/UIO synthesized in the pretest with a 64 mg dosage of Mn(CH_3_COO)_2_·4H_2_O.


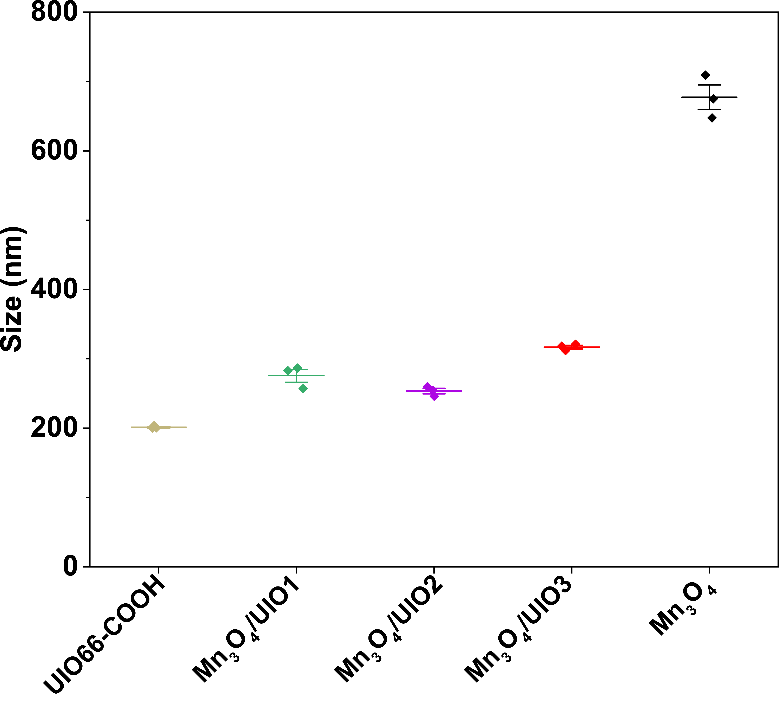


Figure S3. Hydrodynamic diameter of UIO66-COOH(i), Mn_3_O_4_/UIO1 (ii), Mn_3_O_4_/UIO2 (iii), Mn_3_O_4_/UIO3 (iv), and Mn_3_O_4_ NPs (v) via DLS.


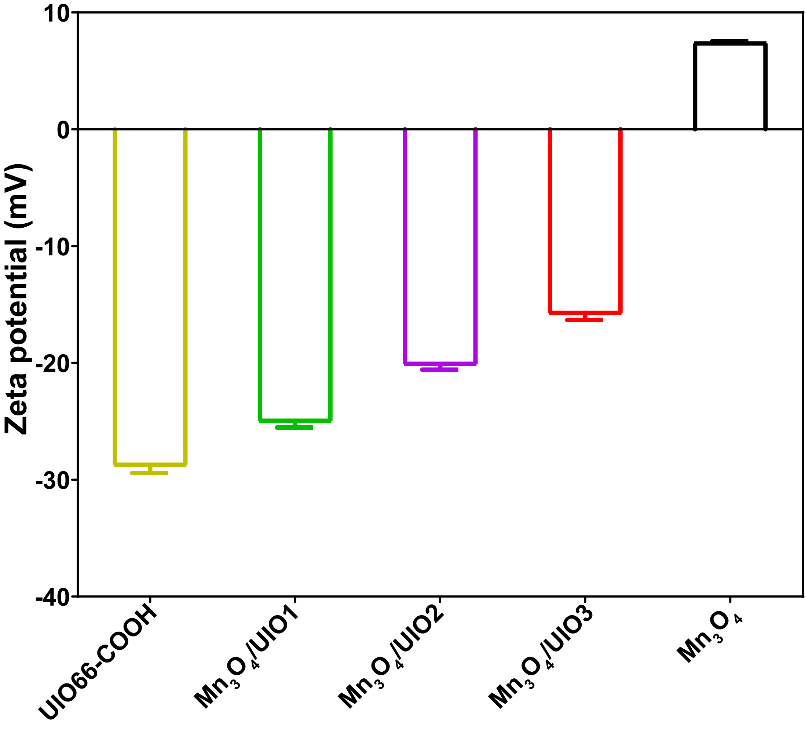


Figure S4. Zeta potential results.


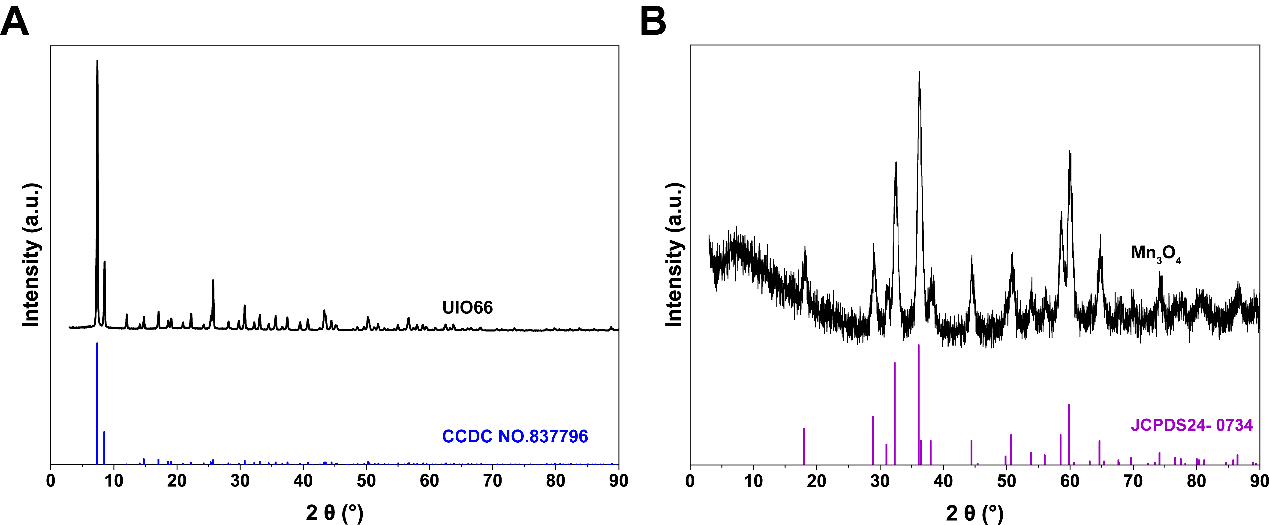


Figure S5. XRD patterns of UIO66 (A) and Mn_3_O_4_ NPs (B), and the corresponding standard patterns.


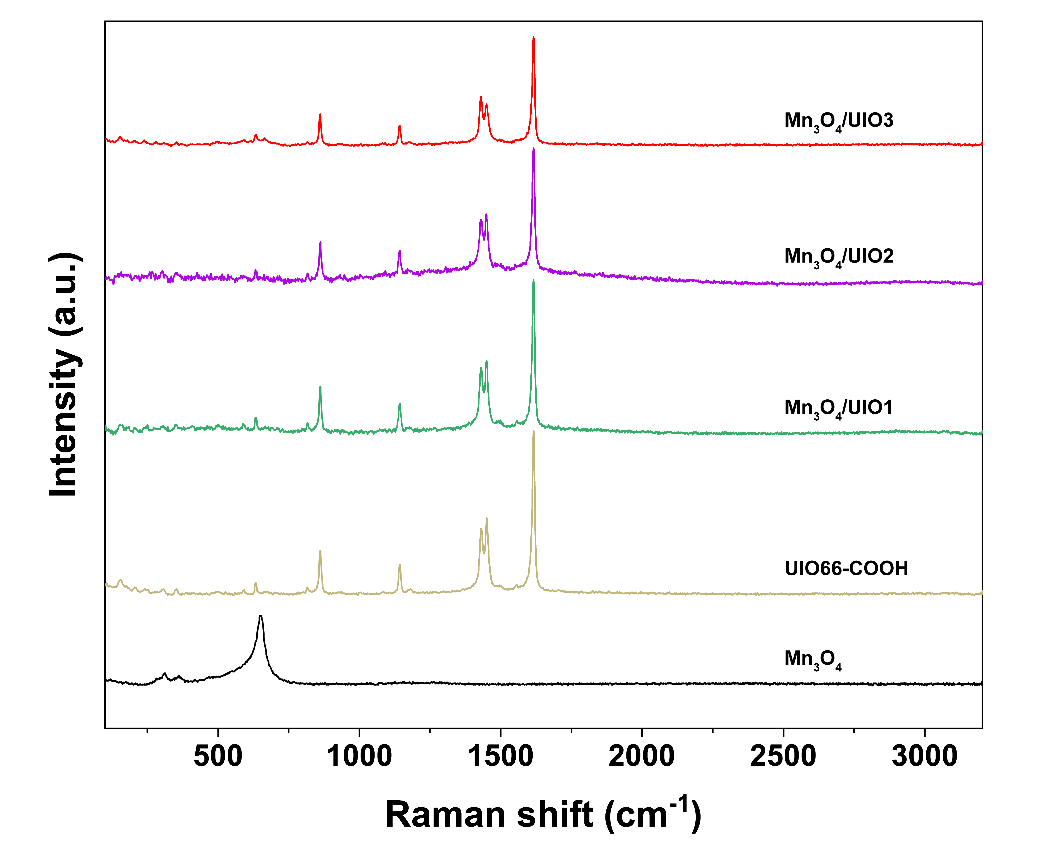


Figure S6. Raman spectra results.


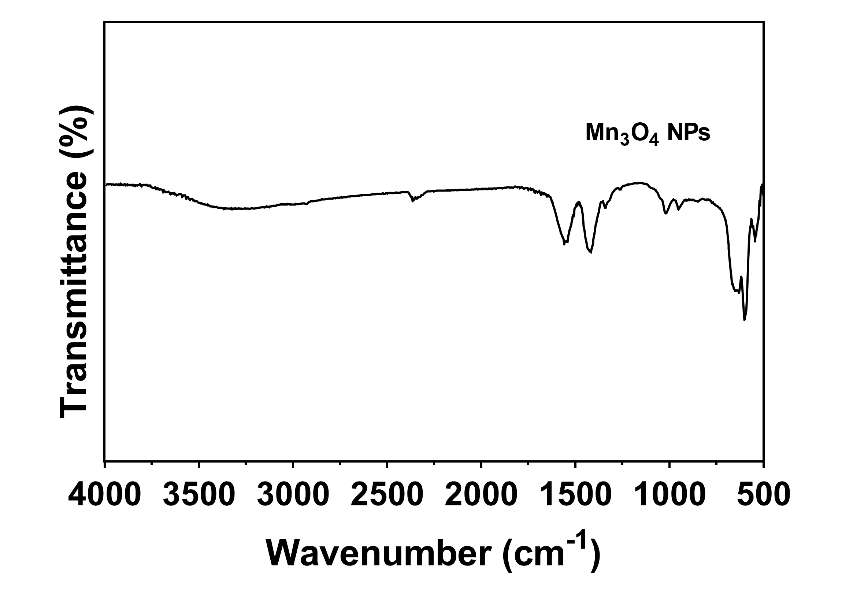


Figure S7. FTIR spectra results of Mn_3_O_4_ NPs.


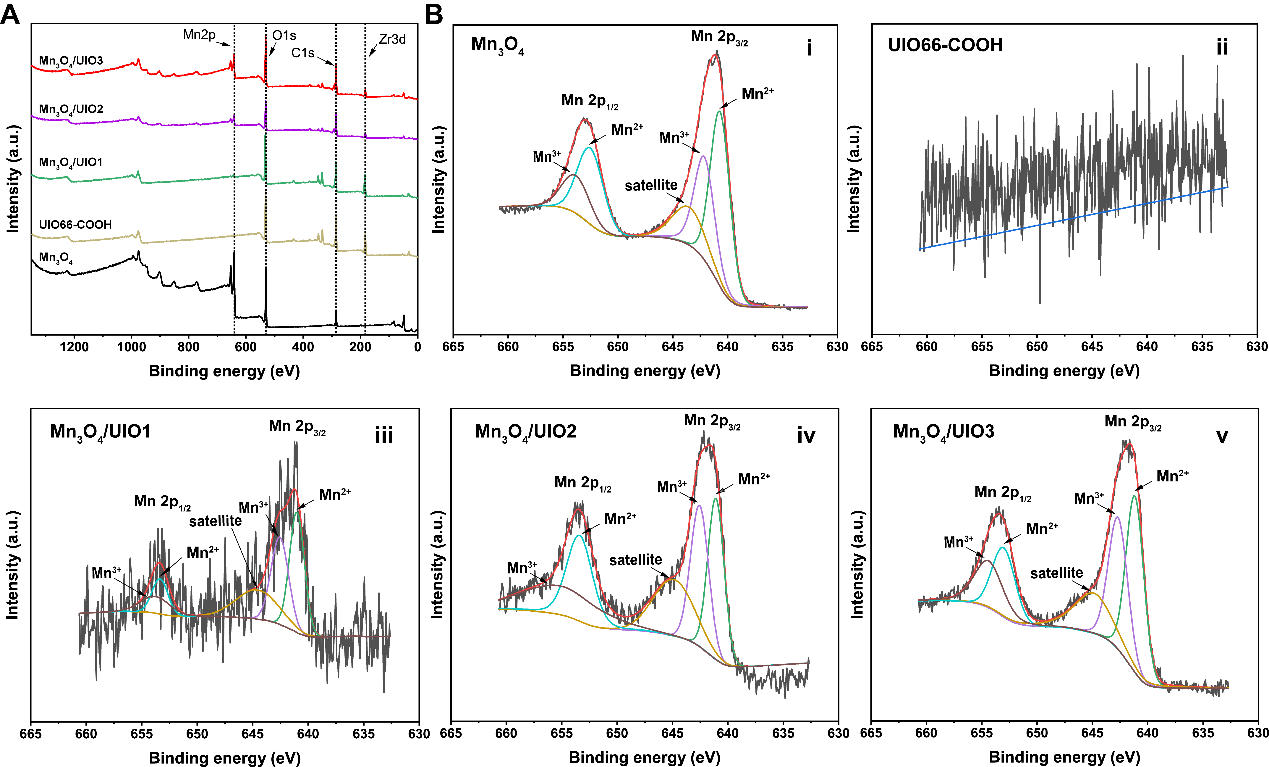


Figure S8. XPS results. (A) XPS full spectrum of Mn_3_O_4_ NPs, UIO66-COOH, Mn_3_O_4_/UIO1, Mn_3_O_4_/UIO2 and Mn_3_O_4_/UIO3. (B) XPS spectrum of the Mn 2p orbital (Mn_3_O_4_ NPs (i), UIO66-COOH (ii), Mn_3_O_4_/UIO1 (iii), Mn_3_O_4_/UIO2 (iv) and Mn_3_O_4_/ UIO3 (v)).


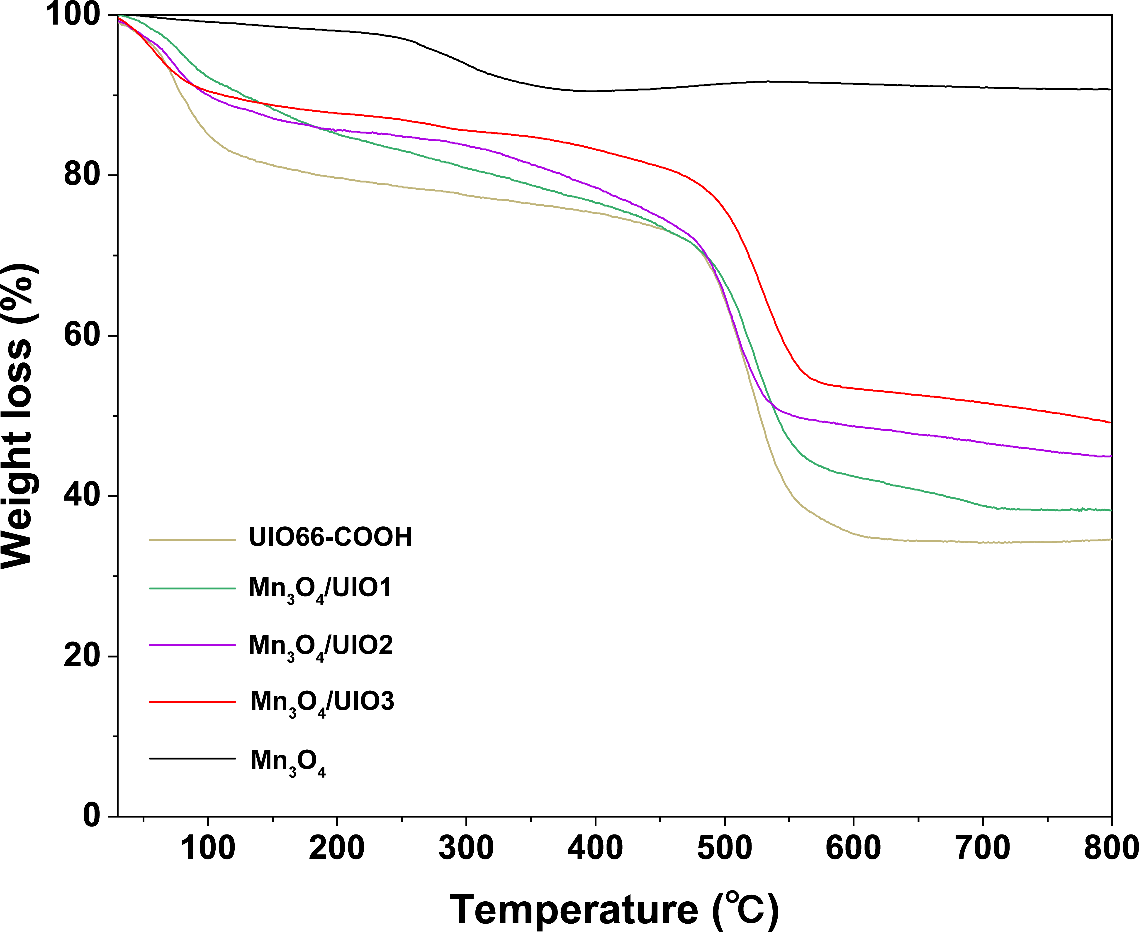


Figure S9. TGA results.


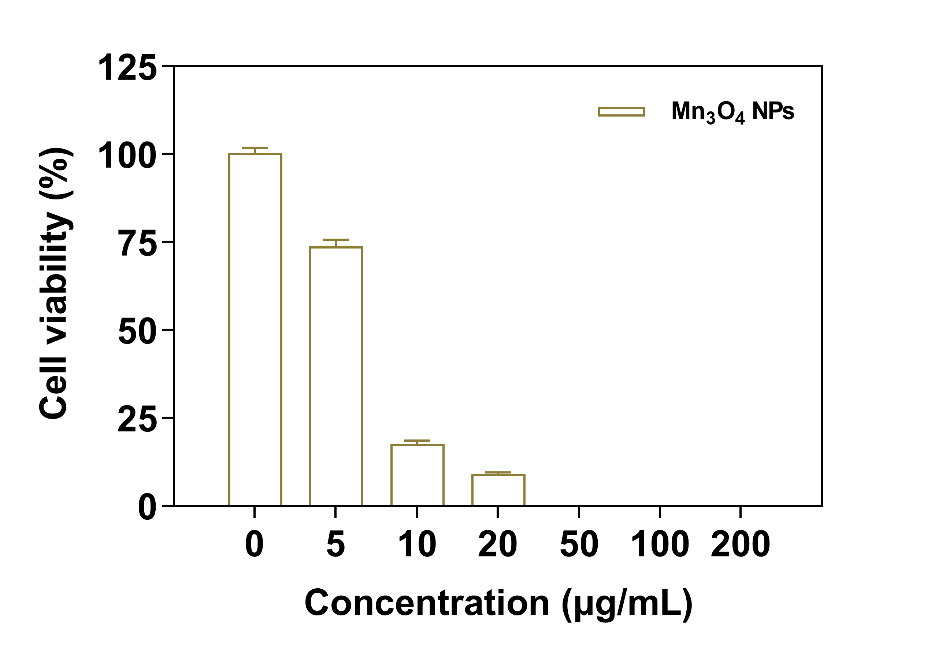


Figure S10. Cytotoxicity *in vitro* of Mn_3_O_4_ NPs by using CCK-8 assay.


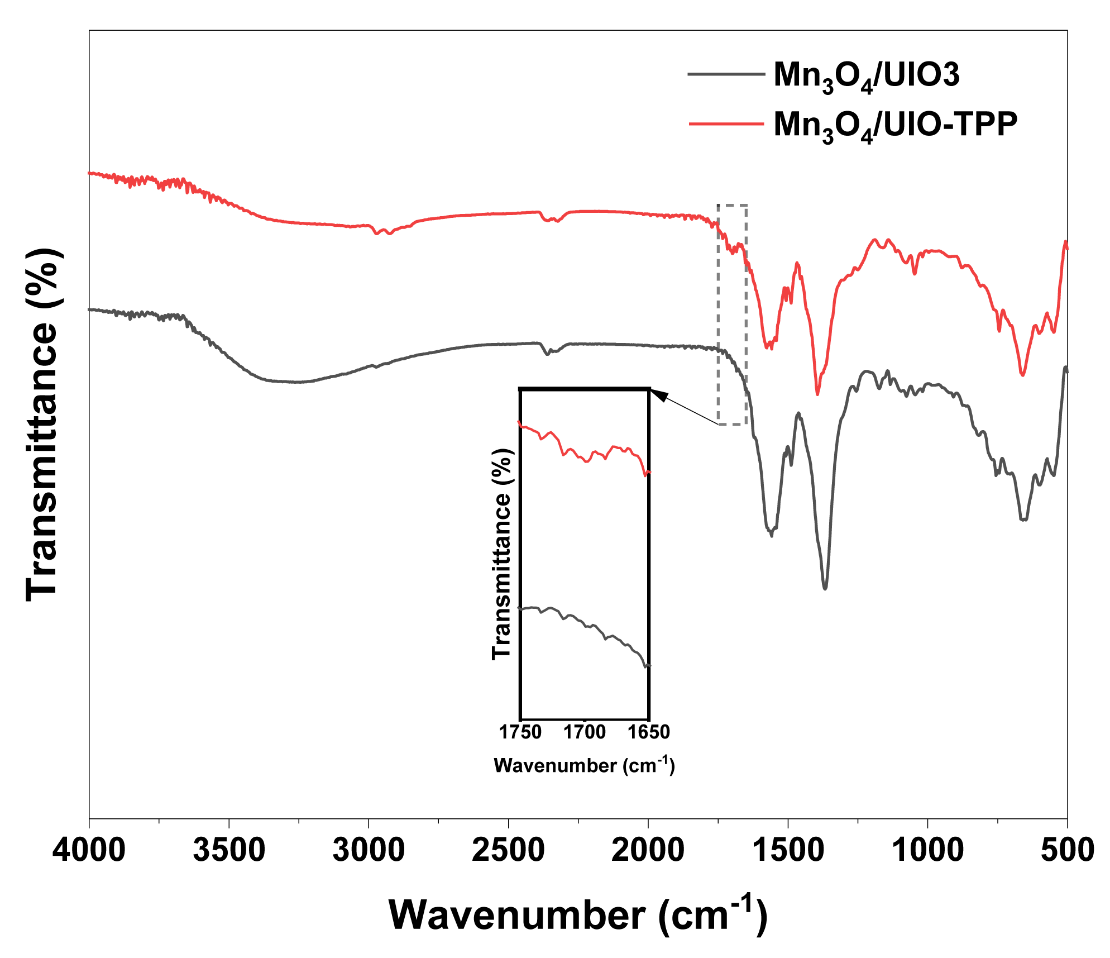


Figure S11. FTIR spectra of Mn_3_O_4_/UIO-TPP and Mn_3_O_4_/UIO3.


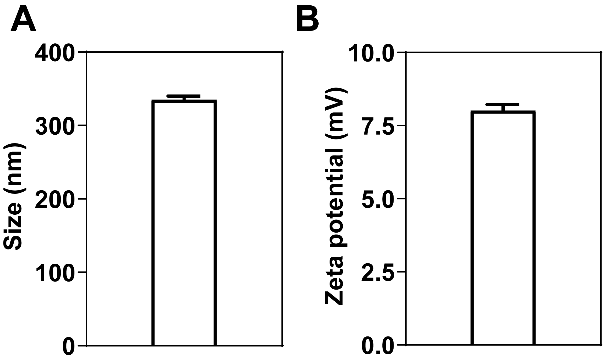


Figure S12. Results of hydrodynamic diameter (A) and zeta potential (B) of Mn_3_O_4_/UIO-TPP nanozyme.


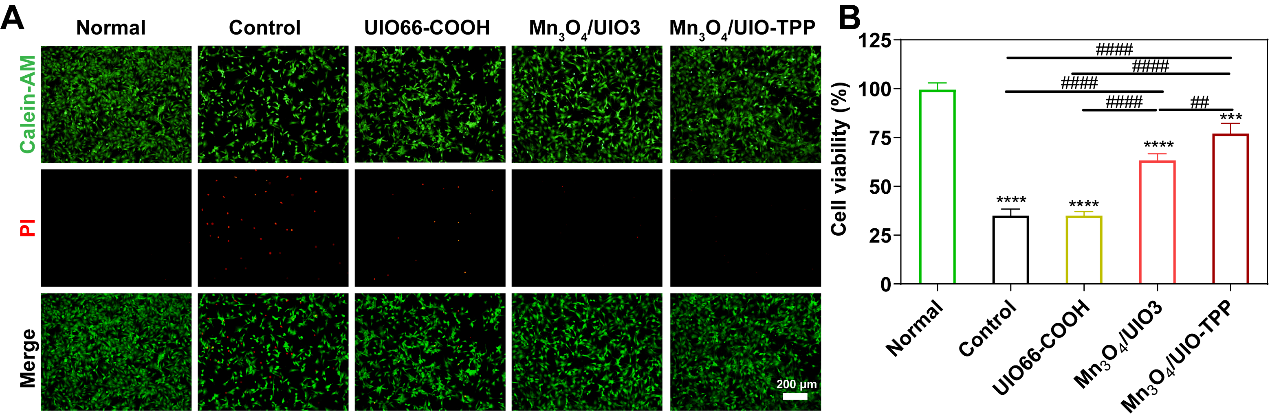


Figure S13. Live and dead staining of chondrocytes-induced by H_2_O_2_ (200 μM, 24 h) after incubated with different samples (20 μg/mL of UIO66-COOH, Mn_3_O_4_/UIO3 and Mn_3_O_4_/UIO-TPP) by a fluorescent microscopy (A) and the corresponding statistical results (B). (‘*’ symbol compared with normal group, ***p < 0.001 and ****p < 0.0001, and ‘#’ symbol compared between two groups, ^##^p < 0.01, ^###^p < 0.001 and ^####^p < 0.0001).


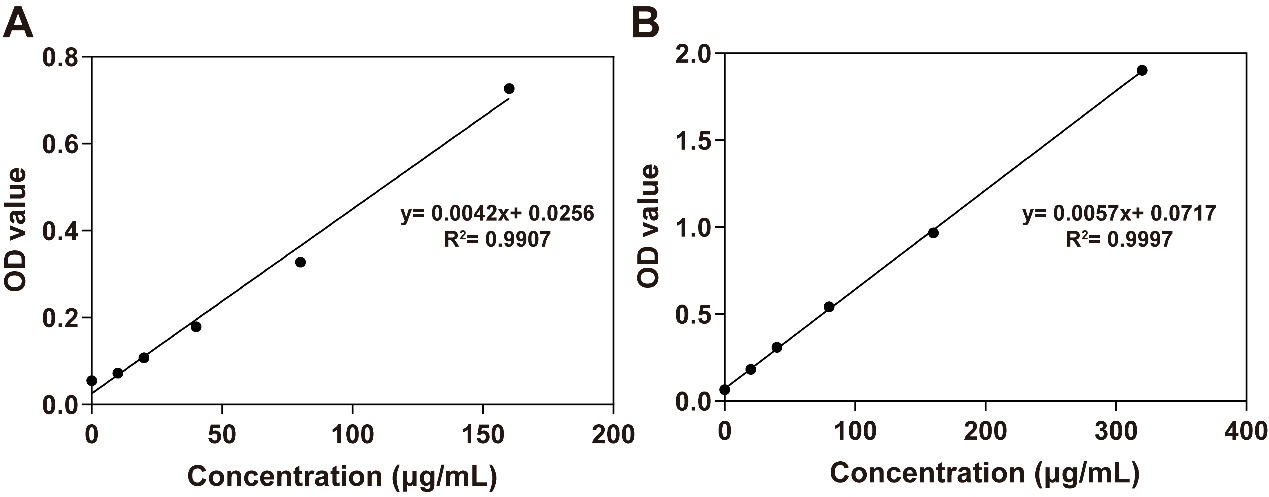


Figure S14. The ELISA standard curves of IL6 (A) and MMP13 (B).


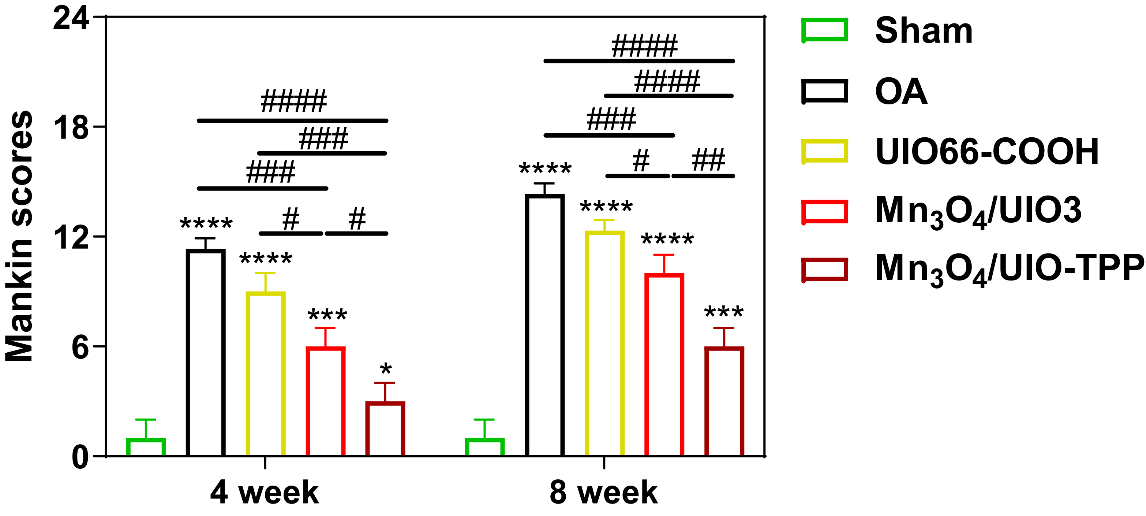


Figure S15. Mankin scores of histological staining in different groups which were collected at 4 and 8 weeks.


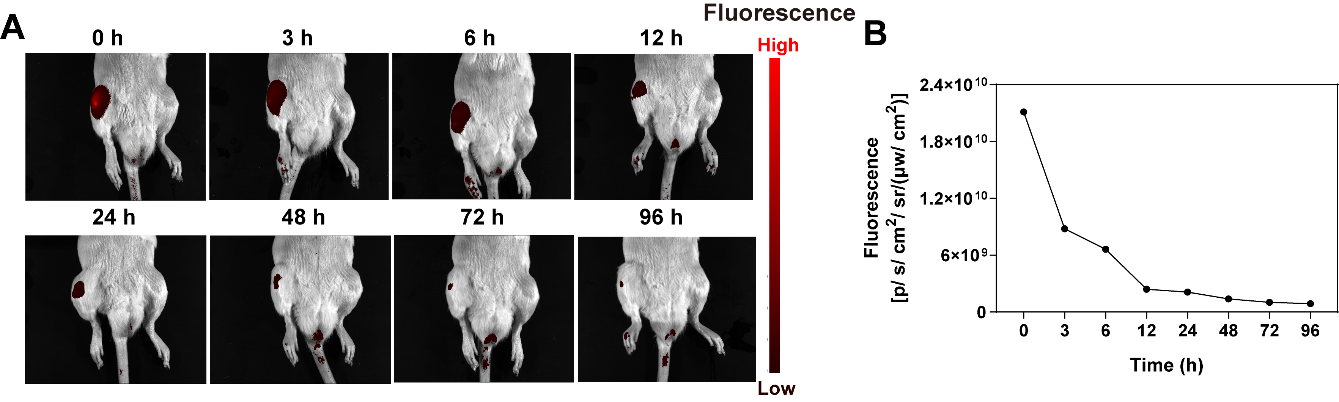


Figure S16. *In vivo* imaging. (A) In vivo biodistribution of Cy5.5-labeled Mn_3_O_4_/UIO-TPP nanozyme in SD rats at different time points and fluorescence intensity results at the indicated time points (B).


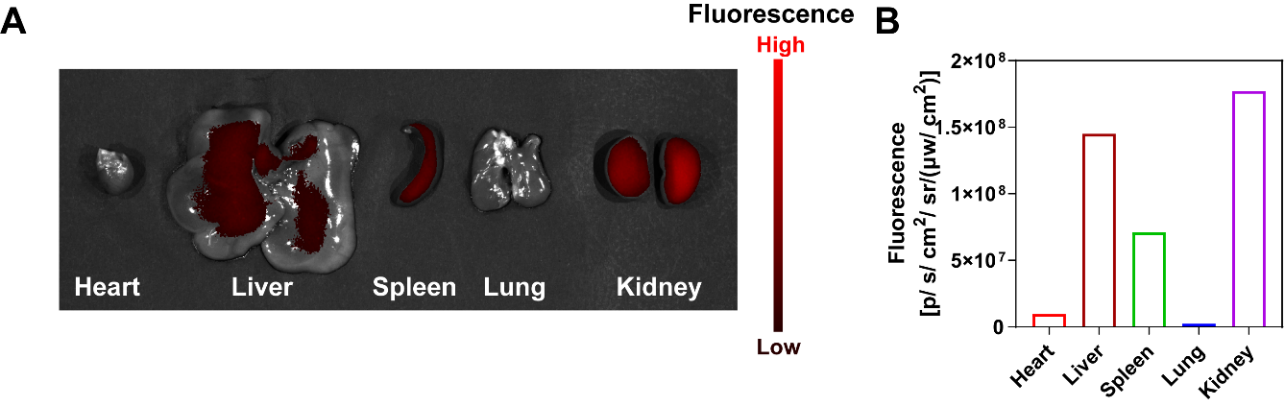


Figure S17. Distribution of Mn_3_O_4_/UIO-TPP nanozyme in tissues of SD rats. (A) The fluorescence image of Cy5.5-labeled Mn_3_O_4_/UIO-TPP nanozyme after 96 h in major organs of SD rats, and fluorescence intensity of measured organs (B).


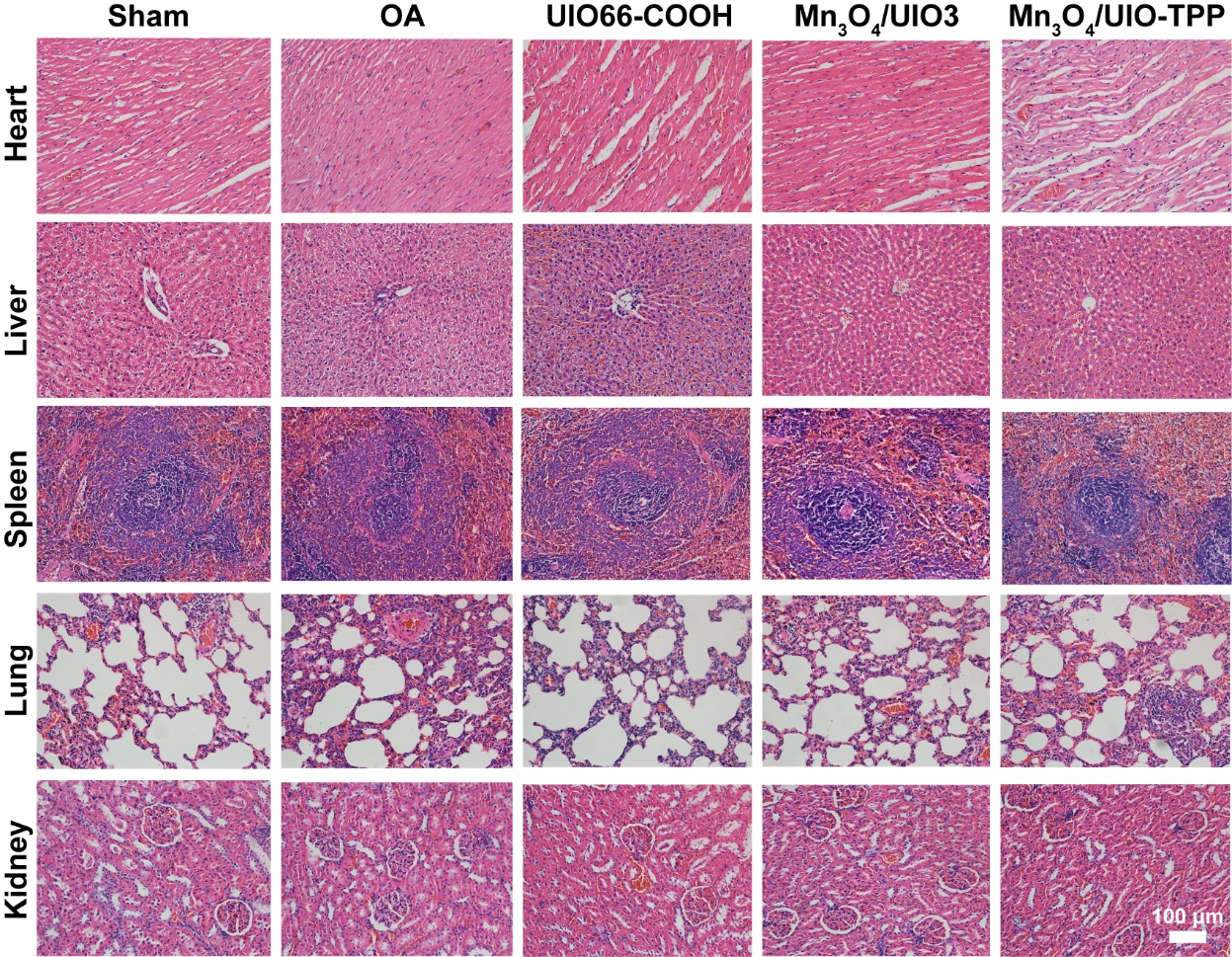


Figure S18. HE images of main organs (Heart, Liver, Spleen, Lung and Kidney) collected at 8 weeks in different treatments.

Table S1 Reaction condition

| Symbol | Mn(CH_3_COO)_2_·4H_2_O (mg) | Ethanol (mL) | UIO66-COOH NPs（mg） | Reaction time（h） |
| --- | --- | --- | --- | --- |
| Mn_3_O_4_/UIO1 | 4 | 20 | 100 | 24 |
| Mn_3_O_4_/UIO2 | 16 | 20 | 100 | 24 |
| Mn_3_O_4_/UIO3 | 32 | 20 | 100 | 24 |

Table S2 Results of N_2_ adsorption-desorption isotherm

| Symbol | BET surface area (m²/g) | | t-Plot micropore area (m²/g) | t-Plot micropore volume (cm³/g) | |
| --- | --- | --- | --- | --- | --- |
| Mn_3_O_4_/UIO1 | | 774.8736 | 735.4255 | | 0.362092 |
| Mn_3_O_4_/UIO2 | | 714.7220 | 674.9045 | | 0.333051 |
| Mn_3_O_4_/UIO3 | | 480.3974 | 421.4932 | | 0.206453 |

Table S3 Element containing via ICP-OES

| Symbol | Zr (wt%) | Mn (wt%) |
| --- | --- | --- |
| Mn_3_O_4_/UIO1 | 23.4175% | 0.7967% |
| Mn_3_O_4_/UIO2 | 20.8115% | 3.3938% |
| Mn_3_O_4_/UIO3 | 20.9974% | 5.1646% |

Table S4 Primer sequence

| Gene | Forward sequence (5’ to 3’) | Reverse sequence (5’ to 3’) |
| --- | --- | --- |
| IL6 | ACAAGTCCGGAGAGGAGACT | ACAGTGCATCATCGCTGTTC |
| MMP13 | ACCATCCTGTGACTCTTGCG | TTCACCCACATCAGGCACTC |
| COX2 | GATGACGAGCGACTGTTCCA | CAATGTTGAAGGTGTCCGGC |
| MMP3 | GGCTGTGTGCTCATCCTACC | TGGAAAGGTACTGAAGCCAC |
| GAPDH | TCCAGTATGACTCTACCCACG | CACGACATACTCAGCACCAG |
| ACTB | ATCCGTAAAGACCTCTATGCCAACA | GGCTACAACTACAGGGCTGACCAC |
| MT-CO2 | CCTCCCATTCATTATCGCCGCCCTTGC | GTCTGGGTCTCCTAGTAGGTCTGGGAA |
